# Analgesic and Functional Outcomes of the Pericapsular Nerve Group Block Versus the Quadratus Lumborum Block After Hip Surgery: A Systematic Review and Meta‐Analysis

**DOI:** 10.1155/prm/7392040

**Published:** 2026-09-06

**Authors:** I-Ning Hsu, Yu-Pin Huang, Yu-Lin Tsai, Wen-Chung Tsai, Yuan-Yang Cheng, Liang-Jun Ou-Yang

**Affiliations:** ^1^ Department of Physical Medicine and Rehabilitation, Chang Gung Memorial Hospital Linkou Center, Taoyuan, Taiwan; ^2^ Department of Anesthesiology, Far Eastern Memorial Hospital, New Taipei City, Taiwan, femh.org.tw; ^3^ Department of Physical Medicine and Rehabilitation, Taichung Veterans General Hospital, Taichung 40705, Taiwan, vghtc.gov.tw; ^4^ Department of Industrial Engineering and Enterprise Information, Tunghai University, Taichung 40704, Taiwan, thu.edu.tw; ^5^ Department of Physical Medicine and Rehabilitation, Taoyuan Chang Gung Memorial Hospital, No. 123 DingHu Rd Guishan Dist, Taoyuan City 333, Taiwan, cgmh.org.tw; ^6^ Department of Medicine, School of Medicine, Chang Gung University, Taoyuan, Taiwan, cgu.edu.tw; ^7^ School of Medicine, National Yang Ming Chiao Tung University, Taipei 11221, Taiwan, nctu.edu.tw; ^8^ Intelligent Long Term Medical Care Research Center, Department of Post-Baccalaureate Medicine, College of Medicine, National Chung Hsing University, Taichung City 40227, Taiwan, nchu.edu.tw; ^9^ Department of Physical Medicine and Rehabilitation, New Taipei Municipal TuCheng Hospital (Built and Operated by Chang Gung Medical Foundation), No. 6 Sec. 2 Jincheng. Rd. Tucheng Dist., New Taipei City 236, Taiwan

**Keywords:** hip surgery, meta-analysis, motor-sparing, pericapsular nerve group block, postoperative analgesia, quadratus lumborum block

## Abstract

**Objective:**

Pericapsular nerve group (PENG) block and quadratus lumborum block (QLB) are regional anesthesia techniques for postoperative analgesia after hip surgery. No meta‐analysis has directly compared these techniques. This systematic review and meta‐analysis compared analgesic, opioid‐sparing, functional, and safety outcomes between PENG block and QLB.

**Methods:**

PubMed, Embase, Cochrane Library, and Web of Science were searched for relevant articles until April 15, 2026. Head‐to‐head randomized controlled trials (RCTs) comparing PENG block with QLB were included. The primary outcome was postoperative pain, and the secondary outcomes were opioid consumption, functional recovery, and adverse events. Pain outcomes were pooled as standardized mean differences and converted to mean differences on a 0–10 scale for comparison with the 1‐point minimal clinically important difference.

**Results:**

Seven RCTs (536 patients) were included. No significant differences were observed between PENG block and QLB across eight pain assessment windows (four time points, at rest and during movement). After pain data conversion, six of eight pooled estimates fell within ±1 point, indicating no clinically important difference. Scores for the remaining two windows (early pain during movement and 24‐h resting pain) were inconclusive because of high heterogeneity and very low certainty evidence. Notably, 24‐h opioid consumption and nausea and vomiting incidence were similar between PENG block and QLB. Narrative synthesis suggested a preliminary, transient motor‐sparing advantage with PENG in some RCTs, requiring confirmation in adequately powered trials.

**Conclusion:**

PENG block and QLB provide comparable analgesia, opioid‐sparing benefits, and short‐term safety after hip surgery. However, the evidence for early pain during movement and 24‐h resting pain exhibited high heterogeneity and very low certainty of evidence and should be interpreted cautiously. A possible early motor‐sparing benefit of PENG block is an exploratory signal and warrants further confirmation in adequately powered trials using objective functional endpoints.

## 1. Introduction

Of late, hip fracture and hip osteoarthritis have become major health concerns worldwide [1, 2]. Surgery is the primary treatment for both conditions. Total hip arthroplasty is the definitive treatment for advanced osteoarthritis and osteonecrosis [3], whereas arthroplasty or internal fixation is performed for hip fractures, depending on the fracture type [4]. With the increasing number of hip‐related procedures, effective management of postoperative pain has become imperative [3].

Although hip surgery is effective, it is associated with considerable postoperative challenges, such as pain, functional impairment, and elevated social burden [5, 6]. Postoperative pain can compromise functional recovery and quality of life [6]. Thus, multimodal pain management strategies have been incorporated into the peri‐ and postoperative care of patients undergoing hip surgery. These strategies include the use of nonsteroidal anti‐inflammatory drugs, acetaminophen, oral and parenteral opioids, and several regional nerve block techniques [7].

Pericapsular nerve group (PENG) block is a regional anesthetic technique used for pain management after hips surgery [8]. First described by Giron‐Arango et al., PENG block targets the articular branches that innervate the anterior hip joint capsule, without blocking motor nerves. It is widely used for hip analgesia and confers several benefits, such as reductions in postoperative pain scores and opioid consumption [8–12].

Recent studies have combined lateral femoral cutaneous nerve (LFCN) block with PENG block [13–16]. The LFCN originates from the lumbar plexus and is a sensory nerve that innervates the anterolateral thigh [17]. Because the skin incision and subcutaneous soft tissue dissection performed during hip surgery often involve the lateral thigh, PENG block plus LFCN block to the PENG block is considered a potentially more effective analgesic strategy [13].

Quadratus lumborum block (QLB), first described by Blanco et al. in 2007, was initially developed to provide analgesia for the abdominal wall [18]. Carney et al. indicated that the injectate administered during QLB longitudinally spreads through the paravertebral space from T5 to L1 and reaches the lumbar plexus and its branches [19, 20]. Clinical data highlight QLB as an effective regional anesthetic technique in abdominal, gynecological, pelvic, and hip procedures [20]. It substantially reduces postoperative pain and opioid consumption [21, 22].

Several clinical trials demonstrated that PENG block outperforms QLB in preserving quadriceps strength during the early postoperative period [11, 15]. However, QLB offers provide wider dermatomal coverage [23]. Therefore, clinicians must decide between prioritizing motor preservation and achieving broader sensory coverage, particularly in older patients for whom early mobilization, fall prevention, and effective pain management are crucial. Although network meta‐analyses have evaluated regional analgesia techniques for hip surgery [24, 25], direct head‐to‐head comparisons of PENG block with QLB are still lacking. To address this research gap, we conducted the present systematic review and meta‐analysis to compare the analgesic and functional outcomes after hip surgery between PENG block and QLB.

## 2. Methods

### 2.1. Designs

This systematic review and meta‐analysis adhered to the Preferred Reporting Items for Systematic Reviews and Meta‐Analyses (PRISMA) guideline [26].

### 2.2. Eligibility Criteria

Eligible studies were selected using the Patient, Intervention, Comparison, Outcomes, and Study (PICOS) framework.

Participant: Adults (age ≥ 18 years) undergoing unilateral or bilateral hip surgery for any indication.

Intervention: PENG block for postoperative management, including studies that combined LFCN block with PENG block.

Comparison: Studies evaluating the efficacy of QLB in postoperative management. Studies using any QLB approach (anterior, lateral, or posterior, classified according to the injection site relative to the quadratus lumborum muscle) were considered eligible.

Outcome: The primary outcome was postoperative pain at rest and during movement, measured using a visual analog scale (VAS) or a numeric rating scale (NRS). Secondary outcomes were postoperative opioid consumption, functional recovery (measured using time to first ambulation and quadriceps strength), and the postoperative complications.

Studies: Randomized controlled trials were included.

We excluded studies that recruited patients aged < 18 years, those that involved patients with preexisting neurological disorders, and those that reported incomplete pain score data.

### 2.3. Identification and Selection of Studies

A comprehensive literature search was conducted across four databases (PubMed, Embase, Cochrane Library, and Web of Science) to identify eligible studies by screening the titles and abstracts. Studies registered on ClinicalTrials.gov were also reviewed. The search terms were as follows: “pericapsular nerve group block,” “PENG block,” “quadratus lumborum block,” “QLB,” “hip surgery,” and “hip arthroplasty.” The titles, abstracts, and subsequently the full texts of potentially eligible records were screened independently and in duplicate by two reviewers (A and B), and a third investigator (F) was consulted to resolve any disagreement. Supporting 1 presents the complete database‐specific search strategies.

### 2.4. Data Extraction and Management

Data on the following patient characteristics were extracted: mean age, sex, American Society of Anesthesiologists (ASA) physical status, anesthesia technique, surgical procedure, and postoperative analgesics regimen. In addition, information on the following intervention characteristics was extracted: block type and approach, surgical technique, and local anesthetic regimen. The following outcome data were obtained: postoperative pain scores, opioid consumption, postoperative complications, time to first ambulation, and quadriceps strength. Table 1 presents a summary of the extracted data.

**TABLE 1:** Characteristics of the included randomized controlled trials (*n* = 7).

Study	Enrolled patients (PENG/QLB) (n)	Surgical indication	Surgical procedure	PENG block	QLB block	Rescue analgesia	Primary outcome	Secondary outcome
Abdelsalam [12] Egypt	35/35 Mean age, 57.0 years ASA I‐III Spinal anesthesia	Mixed (THA/bipolar hemi)	Hip arthroplasty (Approach not mentioned)	PENG alone; bupivacaine 0.25%, 20 mL	Anterior; bupivacaine 0.25%, 20 mL	Morphine IV	Time to first analgesic request	1. Postoperative pain (VAS at 10) 2. Opioid consumption 3. Hip ROM 4. Block‐related complications
Aslan [15] Turkey	40/40 Mean age, 69.3 years ASA I‐III Spinal anesthesia	Hip fracture	Total hip arthroplasty (Approach not mentioned)	PENG + LFCN; bupivacaine 0.25%, 25 + 5 mL	Anterior; bupivacaine 0.25%, 30 mL	Morphine PCA; tramadol	Cumulative 24 h IV morphine	1. Postoperative pain (NRS at 10) 2. Quadriceps strength 3. Patient satisfaction 4. Block‐related complications
Elshall [35] Egypt	31/31 Mean age, 69.8 years ASA I‐III General anesthesia	Hip fracture	Total hip arthroplasty (anterolateral approach)	PENG alone; bupivacaine 0.25%, 30 mL^c^	Anterior; bupivacaine 0.25%, 30 mL^c^	Morphine IV	Total 24 h morphine	1. Postoperative pain (NRS at 10) 2. Quadriceps strength
Et [11] Turkey	30/30 Mean age, 70.4 years ASA I‐III Spinal anesthesia	Mixed (fracture/OA)^a^	Total hip arthroplasty (Posterolateral approach)	PENG alone; bupivacaine 0.5%, 20 mL	Anterior; bupivacaine 0.5%, 30 mL	Oxycodone PO	Highest pain score (NRS) over first 48 h	1. Postoperative opioid consumption 2. Postoperative pain (NRS at 10) 3. Quadriceps strength 4. Quality of recovery
Hay [16] USA	50/51 Mean age, 64.3 years ASA not specified. Spinal anesthesia	Elective OA	Total hip arthroplasty (anterior, posterior, lateral approach)	PENG + LFCN; ropivacaine 0.25%, 20 + 10 mL	Lateral; ropivacaine 0.25%, 30 mL	Hydromorphone IV/oxycodone PO	Cumulative 72 h IV MME	1. Postoperative pain (VAS at 100) 2. Time to first ambulation 3. Patient‐centered outcomes
Şehirlioğlu [36] Turkey	43/30 Mean age, 74.4 years ASA I‐III Spinal anesthesia	Hip fracture	ORIF, IM, or hemiarthroplasty	PENG alone; bupivacaine 0.25%, 20 mL	Anterior; bupivacaine 0.25%, 20 mL	Tramadol IV	Total 48 h tramadol	1. Time to first rescue analgesia 2. Postoperative pain (NRS at 10) 3. Complications
Wang [34] China	45/45 Mean age, 54.1 years ASA I‐III General anesthesia	Elective OA/ONFH/DDH	Total hip arthroplasty (posterolateral approach)	PENG alone; ropivacaine 0.5%, 20 mL	Anterior; ropivacaine 0.33%, 30 mL^b^	Morphine SC	Highest pain score (VAS) reported in the PACU	1. Postoperative pain (NRS at 100) 2. Opioid consumption 3. Quadriceps strength 4. Length of stay in the hospital 5. Complications

*Note:* IV, intravenous; OA, osteoarthritis; ONFH, osteonecrosis of the femoral head; PENG, pericapsular nerve group block; SC, subcutaneous; PACU, postanesthesia care unit.

Abbreviations: ASA, American Society of Anesthesiologists physical status; DDH, developmental dysplasia of the hip; IM, intramedullary nailing; LFCN, lateral femoral cutaneous nerve block; MME, morphine milligram equivalents; NRS, numerical rating scale; ORIF, open reduction and internal fixation; PCA, patient‐controlled analgesia; PO, per os (oral); QLB, quadratus lumborum block; RoB, risk of bias (RoB 2); THA, total hip arthroplasty; VAS, visual analog scale.

### 2.5. Quality of Included Studies

Two reviewers (A and C) independently assessed the risk of bias in the included studies by using the Risk of Bias 2.0 (RoB 2.0) tool recommended by Cochrane Handbook for Systematic Reviews of Intervention [27]. Disagreements between the two reviewers were resolved through discussion with a third reviewer (F). The excluded studies after full‐text review are listed in Supporting 2. The risk of bias diagram is presented in Supporting 3. The certainty of evidence for each outcome was evaluated using the Grading of Recommendations Assessments, Development, and Evaluation (GRADE) framework across five domains: risk of bias, inconsistency, indirectness, imprecision, and publication bias. Table 2 and Supporting 4 present summary judgments and reasons for downgrading.

**TABLE 2:** Summary of findings: pooled effect estimates and certainty of evidence (GRADE) for the comparison of the PENG block versus the QLB after hip surgery.

Outcome (time window)	k	N	Effect estimate (95% CI)	I^2^ (%)	Certainty (GRADE)^a^	Statistical and clinical interpretation^b^
*Postoperative pain—standardized mean difference (SMD; negative favors PENG)*						
Pain at rest, early (∼3 h)	5	386	−0.20 (−0.57 to 0.17)	43.3	Moderate	No significant difference. A clinically important difference is excluded.
Pain on movement, early (∼3 h)	5	386	−0.44 (−1.08 to 0.20)	81.8	Very low	No significant difference. A clinically important difference could not be excluded.
Pain at rest, 12 h	4	296	0.05 (−0.26–0.36)	0	Moderate	No significant difference. A clinically important difference is excluded.
Pain on movement, 12 h	4	296	0.03 (−0.33–0.39)	0	Moderate	No significant difference. A clinically important difference is excluded.
Pain at rest, 24 h	4	314	−0.10 (−0.98 to 0.79)	82	Very low	No significant difference. A clinically important difference could not be excluded.
Pain on movement, 24 h	5	376	0.00 (−0.32 to 0.32)	21	Moderate	No significant difference. A clinically important difference is excluded.
Pain at rest, late (48 h)	3	234	0.13 (−0.01–0.28)	0	Moderate	No significant difference. A clinically important difference is excluded.
Pain on movement, late (48 h)	3	234	0.11 (−0.36–0.58)	0	Moderate	No significant difference. A clinically important difference is excluded.

*Secondary outcomes*						
Opioid consumption, 24 h (SMD; negative favors PENG)	4	313	0.04 (−0.74–0.82)	77.1	Very low	No significant difference
Nausea (RR; < 1 favors PENG)	5	373	0.81 (0.53–1.26)	0	Low	No significant difference
Vomiting (RR; < 1 favors PENG)	4	300	1.07 (0.55–2.07)	0	Low	No significant difference

*Note:* Grading of Recommendations Assessment, Development and Evaluation; *k*, number of trials; *N*, total participants; GRADE.

Abbreviations: CI, confidence interval; MCID, minimal clinically important difference; SMD, standardized mean difference; RR, risk ratio.

^a^Please refer to Supporting 4 for the detailed GRADE appraisal for the certainty of evidence.

^b^For clinical interpretability, SMDs were re‐expressed as mean differences on a common 0–10 pain scale and compared against an MCID of 1 point. The details of the re‐expression of the pooled pain effects are listed in Supporting 7.

### 2.6. Data Synthesis and Statistical Analysis

All analyses were performed in *R* Version 4.5.2 (R Foundation for Statistical Computing, Vienna, Austria) by using the meta (Version 8.3.0) [28] and metafor (Version 5.0.1) [29] packages. Continuous outcomes (postoperative pain and opioid consumption) are presented as standardized mean differences (SMDs) with corresponding 95% confidence intervals (CIs) because the included studies used different scales for outcome assessment. Pooled estimates were calculated using a random‐effects model with the restricted maximum likelihood estimator for interstudy variance, and the CIs were derived using the Hartung–Knapp adjustment method [30], [31]. If an outcome was reported at multiple time points, measurements were grouped into four predefined windows: early (closest to 3 h), 12 h, 24 h, and late (48 h). Resting pain and movement‐induced pain were analyzed separately within each time window. Pain outcomes were pooled as SMDs and converted to mean differences on a 0–10 scale for comparison with the 1‐point minimal clinically important difference (MCID) [32]. Dichotomous outcomes are summarized as risk ratios (RRs) and were pooled using the Mantel–Haenszel common‐effect model [27]. When original data were reported as medians and interquartile ranges, they were converted to means and standard deviations (SDs) by using the approximation recommended in Cochrane Handbook for Systematic Reviews of Intervention [27]. Subgroup analyses by peripheral block regimen (PENG block alone vs. PENG block + LFCN block) and QLB approach were performed. Interstudy heterogeneity was quantified using the Cochrane statistics and Cochran’s *Q* test [33]. Two sensitivity analyses were performed: a reanalysis excluding trials in which SDs were derived through data conversion and a leave‐one‐out analysis. Because < 10 studies were available for each outcome, meta‐regression, funnel plots, and Egger’s tests were not performed. Outcomes that could not be pooled statistically were synthesized qualitatively.

## 3. Results

### 3.1. Study Selection

**FIGURE 1:**
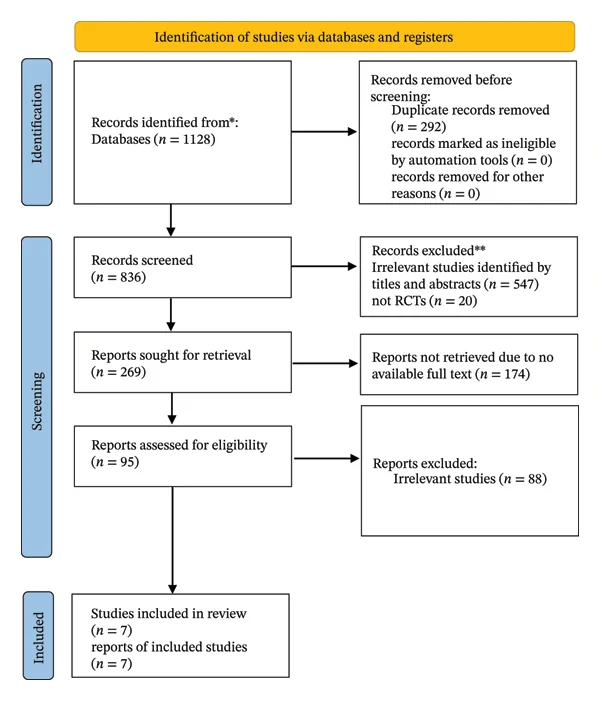
Preferred Reporting Items for Systematic Reviews and Meta‐Analyses (PRISMA) 2020 flow diagram.

The literature search identified 1128 records, of which 292 were duplicates. After duplicate removal, the remaining 836 articles were screened, and 547 irrelevant studies and 20 nonrandomized studies were excluded. After title and abstract screening, the full texts of 95 studies were assessed for eligibility. Ultimately, seven RCTs were included in the analysis [11, 12, 15, 16, 34–36]. Figure 1 presents a flowchart depicting study selection.

### 3.2. Methodology Quality of the Included Studies

**FIGURE 2:**
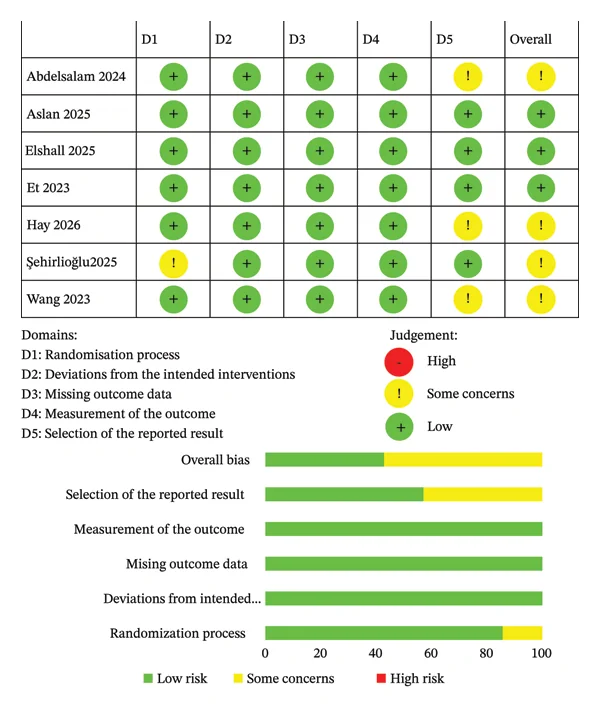
Risk of bias for the included studies assessed using the Risk of Bias 2.0 Tool.

Among the seven RCTs, three were rated as having a “low risk of bias” [11, 15, 35] and four were rated as having “some concerns” [12, 16, 27, 28]. Figure 2 presents the results of study quality assessment.

### 3.3. Characteristics of the Included Studies

This included 536 patients (mean age: 65.05 years; women 55.2%) in this systematic review. Most patients underwent THA, whereas a relatively small population of patients underwent other hip procedures [11, 12, 15, 16, 34–36]. Surgical indications included hip fracture, degenerative diseases, osteonecrosis, and developmental dysplasia. Six studies stratified patients by ASA physical status (I, II, or III) [11, 12, 15, 34–36], whereas the remaining study did not report ASA physical status [16]. Five studies used spinal anesthesia [11, 12, 15, 16, 36], whereas two studies used general anesthesia [34, 35]. Two studies evaluated PENG block plus LFCN block [15, 16], whereas the remaining five studies evaluated PENG block alone [11, 12, 34–36]. Six studies used anterior QLB [11, 12, 15, 34–36], whereas one study used lateral QLB [16]. The local anesthetic regimens consisted of bupivacaine or ropivacaine in volumes of 20–30 mL, and concentrations ranged from 0.25% to 0.5%. Table 1 presents the characteristics of the included studies.

## 4. Outcomes

### 4.1. Primary Outcome: Postoperative Pain

All seven RCTs reported postoperative pain, providing data that could be pooled across eight prespecified analysis windows (four time points, each assessed at rest and during movement). One study reported pain outcomes only in graphic form, whereas the remaining studies reported pain score at rest, during movement, or both. Pain was measured using a 10‐point scale in five RCTs [11, 12, 15, 35, 36] and a 100‐point scale in two RCTs [16, 34]. The assessments were grouped into four prespecified time windows: early (closest to 3 h), 12 h, 24 h, and late (48 h).

Several studies reported lower postoperative pain scores with PENG block than with QLB. Aslan et al. reported lower 24‐h pain scores at rest (2.93 ± 1.14 vs. 4.20 ± 1.54, *p* < 0.001) [15]. Wang reported that the PENG group had lower pain scores at rest at 3 h than did the QLB group (35.0 ± 6.2 vs. 38.1 ± 7.5, *p* = 0.031) [34]. Furthermore, the PENG group had significantly lower pain scores during movement than did the QLB group at both 3 h (47.5 ± 7.0 vs. 55.0 ± 8.8, *p* < 0.001) and 6 h (52.1 ± 8.4 vs. 58.4 ± 8.3, *p* = 0.001) [34]. By contrast, Et et al. reported no significant between‐group differences in pain scores at any time point [11].

**FIGURE 3:**
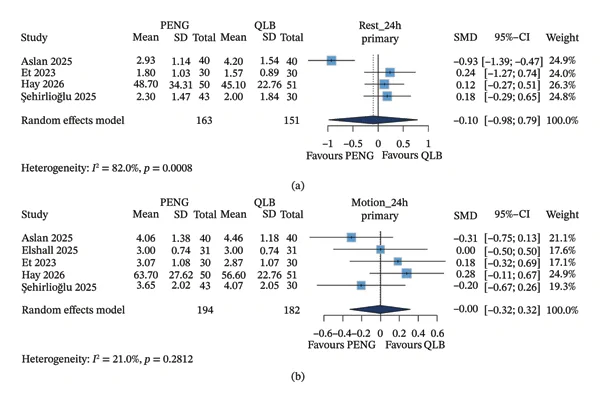
(a) The forest plot compares the postoperative pain at rest at 24 h postoperatively in the PENG group and the QLB group. (b) The forest plot compares the postoperative dynamic pain at 24 h postoperatively in the PENG group and the QLB group.

Figure 3 presents the pooled analysis results for postoperative pain at rest and during movement at 24 h after surgery. Supporting Figure S5‐1 presents the pooled analysis results for the remaining time windows. The forest plot for each window includes the following panels: (a) primary analysis, (b) subgroup analysis by peripheral nerve block regimen (PENG + LFCN vs. PENG alone), (c) subgroup analysis by QLB approach (anterior vs. lateral), (d) sensitivity analysis excluding studies in which SDs were derived through data conversion, and (e) leave‐one‐out analysis. In the primary pooled analysis, postoperative pain scores were comparable between PENG block and QLB groups at all the time windows:

 Early (closest to 3 h), at rest: SMD−0.20 (95% CI−0.57 to 0.17, I^2^ = 43.3%) (Supporting Figure S5‐1.1a)
 Early (closest to 3 h), during movement: SMD−0.44 (95% CI−1.08 to 0.20, I^2^ = 81.8%) (Supporting Figure S5‐1.2a). The subgroup analysis restricted to trials using the PENG block and to the anterior QLB showed that the estimate favored the PENG block (SMD−0.66, 95% CI−1.12 to−0.19, I^2^ = 29.6%) (Supporting Figure S5‐1.2b, S5‐1.2c).
 12 h, at rest: SMD 0.05 (95% CI−0.26 to 0.36, I^2^ = 0%) (Supporting Figure S5‐1.3a)
 12 h, during movement: SMD 0.03 (95% CI−0.33 to 0.39, I^2^ = 0%) (Supporting Figure S5‐1.4a)−24 h, at rest: SMD−0.10 (95% CI−0.98 to 0.79, I^2^ = 82%) (Figure 3a) and Supporting Figure S5‐1.5a). In the leave‐one‐out analysis omitting Aslan, the estimate became 0.16 (95% CI 0.02 to 0.30, I^2^ = 0%) (Supporting Figure S5‐1.5e)
 24 h, during movement: SMD 0.00 (95% CI−0.32 to 0.32, I^2^ = 21%) (Figure 3b and Supporting Figure S5‐1.6a)
 Late (48 h), at rest: SMD 0.13 (95% CI−0.01 to 0.28, I^2^ = 0%) (Supporting Figure S5‐1.7a)
 Late (48 h), during movement: SMD 0.11 (95% CI−0.36 to 0.58, I^2^ = 0%) (Supporting Figure S5‐1.8a)

The results of subgroup analyses by the peripheral nerve block regimen and by QLB approach are presented in panels (b) and (c), respectively, of each window‐specific forest plot (Supporting Figure S5‐1.1b, c to S5‐1.8b, c). For early pain at rest, the prespecified primary analysis revealed no significant between‐group difference. A small but significant effect favoring PENG block was observed only after the exclusion of two studies in which SDs were derived through data conversion [16, 35], with no residual heterogeneity (SMD −0.41, 95% CI −0.79 to −0.03, I2 = 0%, Supporting Figure S5‐1.1d). For early pain during movement, the two subgroup analyses (restricted to the PENG block alone and to anterior QLB) and the sensitivity analysis (excluding studies in which SDs were derived through data conversion) comprised the same set of trials. All analyses revealed a significant benefit with PENG block (SMD ‐0.66, 95% CI ‐1.12, −0.19; Supporting Figure S5‐1.2 b to 5‐1.2 days). For 24‐h resting pain, the leave‐one‐out analysis of 24‐h resting pain revealed that excluding the study by Aslan et al. eliminated the observed heterogeneity (I2 = 0%; Supporting Figure S5‐1.5e). The significant findings did not rule out a clinically important difference (Supporting Figure S7.2b and S7.2c).

### 4.2. Secondary Outcome: Opioid Consumption

**FIGURE 4:**
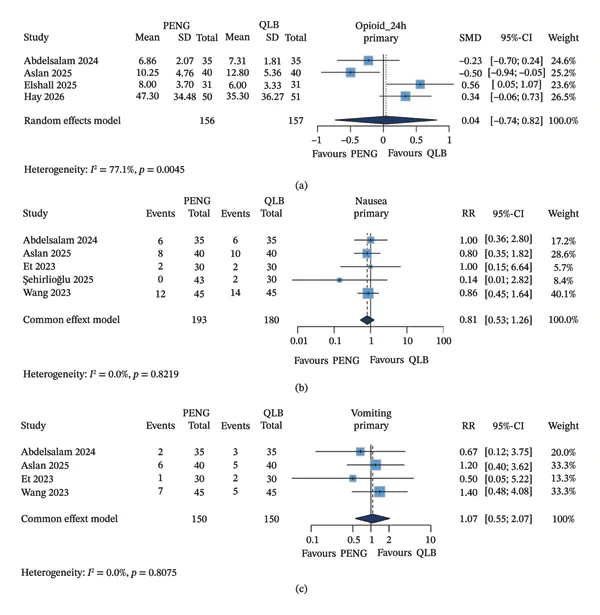
Secondary outcomes. (a) The forest plot compares the total opioid consumption at 24 h postoperatively in the PENG group and the QLB group. (b) The forest plot compares the number of nausea events postoperatively in the PENG group and the QLB group. (c) The forest plot compares the number of vomiting events postoperatively in the PENG group and the QLB group.

Opioid consumption could be pooled only for the 24‐h postoperative time point because sufficient data were unavailable for the other time points. The types of opioids and their routes of administration varied among the included studies. Aslan reported lower 24‐h opioid consumption in the PENG group than in the QLB group (10.25 ± 4.76 vs. 12.80 ± 5.36 mg IV morphine; *p* = 0.027) [15]. By contrast, Elshall et al. and Şehirlioğlu et al. reported lower rescue opioid consumption in the QLB group than in the PENG group (*p* = 0.035 and *p* = 0.004, respectively) [35, 36]. Hay et al. reported higher cumulative opioid consumption in the PENG group than in the QLB group at later cumulative time points (48–72 h; adjusted *p* ≤ 0.01) [16]. The remaining trials reported no significant between‐group difference. The pooled analysis results for 24‐h opioid consumption (*k* = 4) did not differ between the PENG and the QLB groups (SMD 0.04, 95% CI−0.74 to 0.82; I^2^ = 77% (Figure 4a and Table 2). The subgroup and sensitivity analyses yielded consistent results (Supporting Figure S5‐2.1b to S5‐2.1).

### 4.3. Functional Outcomes

Functional outcomes were reported inconsistently across the included studies. Hence, the data were not pooled; rather, the evidence was synthesized narratively (Table 3).

**TABLE 3:** Narrative summarization of functional outcomes.

Outcome/study	Definition and measure (unit; data form)	PENG	QLB	*p* value	Direction/note
*Ambulation*					
Et [11]	Time to first mobilization (h; mean ± SD) Definition: definition not stated.	13.17 ± 4.43	17.30 ± 4.92	0.003	Favors PENG (earlier)
Elshall [35]	Time to first walk (h; median [Q1–Q3]) Definition: ≥ 3 steps with a walker, timed from completion of surgery.	8.0 (8.0–12.0)	24 (18.0–24.0)	< 0.001	Favors PENG (earlier)
Abdelsalam [12]	Time to walk (h; mean ± SD) Definition: able to walk ≥ 3 steps with a walker.	21.6 ± 1.65	20.97 ± 1.34	0.084	No difference
Hay [16]	Time to first ambulation (min; median [IQR]) Definition: definition not stated.	465 (128)	456 (79)	0.185	No difference
Hay [16]	Distance ambulated (feet; median [IQR]) Definition: definition not stated.	200 (150)	180 (200)	0.304	No difference

*Quadriceps strength and motor function*					
Et [11]	Quadriceps motor status at 3 h (*n* by category: normal/paresis/paralysis) Definition: 3‐category 0–2 scale—normal (extension against resistance)/paresis (flexion against gravity, not resistance)/paralysis (no extension); does not span a full 0–5 MMT.	9/14/7	5/6/19	0.007	Favors PENG (more motor‐sparing)
Et [11]	Quadriceps motor status at 6 h (*n* by category: normal/paresis/paralysis) Definition: as above.	18/10/2	9/14/7	0.053	No difference (3‐arm *p*)
Elshall [35]	Quadriceps strength, MMT 0–5 (median [Q1‐Q3]); serial 30 min–24 h (value at 6 h) Definition: MMT graded 0–5.	4.0 (4.0–4.0)	2.0 (2.0–3.0)	< 0.001	Favors PENG at 30 min–18 h; equal at 24 h (*p* = 0.368)
Aslan [15]	Quadriceps weakness at 6 h (n/N) Definition: Threshold of weakness not defined in the report.	0/40	6/40	0.026	Favors PENG (less weakness)
Aslan [15]	Quadriceps weakness at 12 h and 24 h (n/N) Definition: as above.	0/40	0/40	—	No difference
Wang [34]	Quadriceps weakness, any (n/N) Definition: weakness = MMT level 0–2 on a 0–5 scale (vs contralateral leg).	0/45	0/45	—	No difference
Şehirlioğlu [36]	Quadriceps weakness, any (n/N) Definition: Threshold of weakness not defined in the report.	0/43	0/30	—	No difference
Hay [16]	Time to return of motor function (min; median [IQR]) Definition: definition not stated.	222 ± 73	221 ± 81	0.768	No difference

Abbreviation: MMT, manual muscle test.

Time to first ambulation was reported in four RCTs, although the definitions varied. Abdelsalam et al. and Elshall et al. defined first ambulation as walking at least three steps with the help of a walker, whereas Et et al. and Hay et al. did not specify the definition of the first ambulation [11, 12, 16, 35]. Two studies favored PENG block over QLB (Et et al., 13.17 ± 4.43 vs. 17.30 ± 4.92 h, *p* = 0.003; Elshall et al., 8 vs. 24 h, *p* < 0.001), whereas the other two reported no significant between‐group difference (Abdelsalam et al., 21.6 ± 1.65 vs. 20.97 ± 1.34 h, *p* = 0.084; Hay et al., 465 vs. 456 min, *p* = 0.185).

Two studies consistently reported that PENG block outperformed QLB in preserving quadriceps strength and motor function during the early postoperative period. Et et al. reported that the proportion of patients with preserved quadriceps function at 3 h was higher in the PENG group than in the QLB group (pairwise test *p* = 0.007), although the difference became nonsignificant at 6 h. Elshall et al. reported that patients’ score in quadriceps manual muscle testing conducted between 30 min and 18 h after surgery were higher in the PENG group than in the QLB group (*p* < 0.001 at multiple time points), but the between‐group difference resolved by 24 h (*p* = 0.368) [35]. Aslan et al. reported that transient quadriceps weakness, an adverse event, at 6 h was more common in the QLB group than in the PENG group (Aslan, *p* = 0.026), but the difference resolved by 12 h (Table 3) [15].

### 4.4. Adverse Events

Adverse events were infrequent and were similar between the PENG group and the QLB group (Supporting 6). Postoperative nausea and vomiting were pooled for meta‐analysis. The pooled risk ratio was 0.81 (95% CI 0.53–1.26; I^2^ = 0%) for nausea and 1.07 (95% CI 0.55–2.07; I^2^ = 0%) for vomiting. Neither outcome differed significantly between the two groups (Figure 4b,c and Table 2). Other events were reported infrequently and included block failure (Elshall et al., 0/31 vs. 0/31) and pruritus (Aslan et al., 2/40 vs. 3/40) [15, 26]. Information of transient quadriceps weakness is presented in the previous section. No falls were reported in any of the included studies.

### 4.5. Certainty of Evidence (GRADE)

Table 2 and Supporting 4 present the results of certainty‐of‐evidence analysis for each outcome. For postoperative pain, scores during most time windows were rated as having moderate‐certainty evidence and were downgraded by one level because of risk of bias. The evidence for 24‐h resting pain and early pain during movement was rated as having very low certainty because it was further downgraded for serious inconsistency (I^2^ > 75%) and imprecision (the converted mean difference CI crossed the MCID). The certainty of evidence for opioid consumption was rated as very low because of downgrades for risk of bias, inconsistency, and imprecision. For adverse events, the certainty of evidence for both postoperative nausea and vomiting were rated as low because of downgrades for risk of bias and imprecision.

## 5. Discussion

This systematic review and meta‐analysis of seven RCTs suggests that PENG block and QLB provide clinically comparable analgesia after hip surgery. Across all eight prespecified pain assessment windows, the primary analyses revealed no significant differences in postoperative pain between the two techniques. When the pooled estimates were converted to mean differences on the 0–10 scale, six of the eight estimates remained within ± 1 point, indicating no clinically important difference. Scores for the remaining two windows, early pain during movement and 24‐h resting pain, surpassed the 1‐point threshold but exhibited substantial heterogeneity and were rated as very‐low certainty evidence. Although QLB provides wider dermatomal coverage than does PENG block, this theoretical advantage did not translate into superior postoperative analgesia. The two groups were similar in terms of 24‐h opioid consumption and the incidence of postoperative nausea and vomiting. Although none of the prespecified primary analyses showed a significant between‐group difference, two signals favoring the PENG block emerged from nonprimary analyses; however, both were supported by limited evidence. First, a significant reduction in early pain during movement was observed only in the nonprimary analyses: the subgroups restricted to studies using PENG block alone and to anterior QLB, and the sensitivity analysis excluding studies with converted SDs (SMD = −0.66, 95% CI−1.12 to−0.19). All three analyses comprised the same four trials and therefore do not represent independent lines of evidence. A similar effect was noted for early pain at rest after the exclusion of two studies in which SDs were derived through data conversion (SMD = −0.41, 95% CI−0.79 to−0.03). Neither early pain during movement nor early pain at rest remained clinically important after data conversion against the 1‐point MCID. Second, an early motor‐sparing effect consistently favored PENG block but could only be synthesized narratively. Therefore, both findings should be considered hypothesis‐generating. They do not alter the overall conclusion of clinical comparability between the two techniques. Any meaningful difference between the two blocks, if exists, most likely lies in early motor function rather than in analgesia.

Regional analgesic techniques have been evaluated in two network meta‐analyses [24, 25]. Hayashi et al. focused on preoperative pain management in patients with hip fractures. Wan et al. highlighted PENG block plus periarticular infiltration as the most effective technique for pain control during the first 24 after surgery. However, Wan et al. included only two trials that directly compared PENG block with QLB, and the treatment rankings were largely based on indirect comparisons [11, 34].

Our review differs from previous network meta‐analyses in four aspects. First, we included only head‐to‐head RCTs, thereby avoiding the assumptions inherent to indirect comparisons. Second, we included three latest trials [16, 35, 36], thereby expanding the available direct comparative evidence. Third, we performed subgroup analyses by peripheral nerve block regimen (PENG block alone vs. PENG block + LFCN block) and QLB approach (anterior vs. lateral), thereby explicitly addressing technical heterogeneity, which was not examined in previous syntheses. Finally, we evaluated each pooled estimate against a prespecified MCID and examined its robustness by using appropriate sensitivity analysis (leave‐one‐out analysis and reanalysis excluding studies in which SDs were derived through data conversion). Therefore, our conclusions are based on not only statistical estimate but also clinical relevance and the robustness of the available evidence.

The clearest difference between the two techniques was observed in early motor function, which is central to the rationale for PENG block. PENG block delivers local anesthetics around the articular branches of the anterior hip capsule, providing effective pain control while sparing the motor fibers of the femoral nerve. Therefore, it is therefore expected to preserve quadriceps strength [37]. By contrast, QLB produces analgesia by spreading the injectate to the lumbar plexus, which may also involve motor fibers [38, 39]. Three RCTs reported findings consistent with this proposed motor‐sparing mechanism during the early postoperative period. Et et al. reported that the proportion of patients with preserved quadriceps motor function at 3 h was higher in the PENG group than in the QLB group, although the between‐group difference resolved by 6 h [11]. Elshall et al. reported that patients’ scores in quadriceps muscle tests conducted between 30 min and 18 h after surgery were higher in the PENG group than in the QLB group, but the between‐group difference resolved by 24 h [35]. Aslan et al. reported that transient quadriceps weakness at 6 h was more common in the QLB group than in the PENG group, but the between‐group difference resolved by 12 h. Early mobilization with the PENG block was reported in two trials [11, 35], but not in two others [12, 16]. The remaining trials reported no weakness in either group.

Opioid consumption and adverse events did not differ significantly between the two techniques. Specifically, 24‐h opioid consumption was similar between the PENG group and the QLB group. However, this outcome was highly heterogeneous because the included studies used different opioid agents, administration routes, and rescue analgesia protocols. Therefore, the certainty of evidence for this outcome was rated as very low. The incidence of postoperative nausea and vomiting was similar between the two groups, with no apparent heterogeneity. Other adverse events were uncommon, and no block failures or falls were reported. Overall, the two techniques had similar short‐term safety profiles, except for transient quadriceps weakness.

Subgroup analyses were performed by separating PENG block alone from PENG block plus LFCN block, and anterior QLB from lateral QLB because these techniques are not mechanistically equivalent. The addition of LFCN block extends cutaneous coverage of the anterolateral thigh, complementing the analgesic effect of PENG block [40]. The anterior and lateral QLB approaches differ in terms of injectate spread and lumbar plexus involvement [38, 39]. However, these factors could not be independently analyzed in this review. PENG block plus LFCN block was evaluated by Aslan et al. [15] and Hay et al. [16]. Notably, the study by Hay et al. [16] was the only trial to use lateral QLB, and the study by Aslan et al.[15] was the only trial to evaluate PENG + LFCN block in an acute hip fracture population. The other trial [16] evaluated PENG + LFCN block in patients undergoing elective surgery for osteoarthritis. The combination of high baseline pain in an acute hip fracture population and additional anterolateral cutaneous coverage by LFCN block may explain the outlying effect observed in our leave‐one‐out analysis of 24‐h resting pain [15, 16]. Thus, the outcomes of peripheral nerve block regimen (PENG block or PENG + LFCN block) and QLB (anterior or lateral) overlapped almost completely. Each subgroup included only one or two trials, making interaction tests difficult to interpret and meta‐regression infeasible. Because lateral QLB was evaluated in only one trial, which also evaluated PENG + LFCN block, the independent effect of this approach on the comparison could not be isolated.

The present study has several limitations. First, the sample size was small (seven RCTs), and several subgroup analyses included one or two trials. Because < 10 trials were available for each outcome, funnel plot analyses could not be performed, and publication bias could not be formally assessed. The small sample size also limited the statistical power of heterogeneity tests, resulting in wide and conservative CIs despite the use of the Hartung–Knapp statistical adjustment. Second, some studies reported outcomes as medians and interquartile ranges, which were converted to means and SDs before data pooling. Third, the RCTs included a mixed population of patients with hip fractures and patients undergoing elective surgery for osteoarthritis; this inconsistency may have contributed to the observed heterogeneity. Individual comorbidities were not consistently reported and thus could not be analyzed. Furthermore, because < 10 trials were available for each outcome, the potential effect of these factors could not be examined using meta‐regression. Fourth, the RCTs used different local anesthetic regimens—bupivacaine or ropivacaine in varying volumes at concentrations ranging from 0.25% to 0.5%. This pharmacological variation may have contributed to the observed heterogeneity. Finally, the risk of bias in the included studies ranged from low risk to some concerns, and the certainty of evidence ranged from moderate to very low. The findings of this review should therefore be interpreted very cautiously.

For clinical implication, both PENG block and QLB are reasonable choices, offering comparable analgesia, opioid‐sparing benefits, and short‐term safety. However, the certainty of evidence for early pain during movement and 24‐h resting pain was rated as very low because of serious inconsistency; therefore, corresponding findings should be interpreted cautiously. A tentative benefit of motor function preservation and early mobilization could be observed in the PENG group; however, the effect appeared to be early and transient. Therefore, the findings should be considered hypothesis‐generating rather than practice‐changing. Because the two techniques appear to be largely interchangeable with respect to analgesia, a key question is whether they differ in their contributions to motor preservation and early mobilization. Therefore, future studies should focus on quantifying motor‐sparing effects by using objective measures, such as hand‐held dynamometry, and assess functional recovery by using a standardized definition of time to first ambulation and validated outcome measures, such as the Timed Up and Go test and the 5‐m walk test. Prespecifying MCIDs for these functional outcomes may help determine whether significant differences in analgesic outcomes translate into clinically meaningful benefits. Future studies should also prespecify and stratify analyses by peripheral nerve block regimen and QLB approach because the present study could not distinguish the effects of PENG block from PENG block plus LFCN block or the effects of anterior QLB from those of lateral QLB. The overlapping effects limits interpretation of both the analgesic and the motor outcomes.

From a practical perspective, the choice between PENG block and QLB need not be based on analgesic superiority because the two techniques demonstrated comparable outcomes across all pain assessment windows, opioid consumption, and short‐term adverse events. Instead, the decision may be guided by operator expertise, institutional familiarity, and patient‐specific considerations. In patients for whom early mobilization is a priority, patients with frailty, and patients with elevated fall risk, PENG block may be considered because of its early and transient motor‐sparing effect. For clinicians who are more experienced with QLB, it remains an equally reasonable option. This guidance is pragmatic rather than strictly evidence‐based and should be revisited as additional objective data on functional outcomes become available.

## 6. Conclusion

In patients undergoing hip surgery, both PENG block and QLB provide comparable analgesia, opioid‐sparing benefits, and short‐term safety, Thus, the choice between the two techniques can be guided by clinical judgment and operator familiarity. Current evidence suggests the PENG block may better preserve early motor function than QLB, but this signal is preliminary and requires confirmation. Future RCTs should evaluate this potential advantage by using objective measures of quadriceps strength and functional recovery and should prespecify and stratify analyses by peripheral nerve block regimen and QLB approach.

## Funding

This study received no funding or support.

## Conflicts of Interest

The authors declare no conflicts of interest.

## Supporting Information

Additional supporting information can be found online in the Supporting Information section.

## Supporting information


**Supporting Information** Supporting Materials Supporting 1 describes the searching strategies, including search terms, search combinations, and the number of studies retrieved. Supporting 2 lists the studies excluded after full‐text screening. Supporting 3 presents the risk of bias assessment of each included studies using the Cochrane Risk of Bias 2.0 tool. Supporting 4 presents the appraisal of the included studies using the GRADE tool Supporting 5 presents the results of the meta‐analyses, including the forest plots, the subgroup analyses, and the sensitivity analyses.

## Data Availability

The data that support the findings of this study are available on request from the corresponding author. The data are not publicly available due to privacy or ethical restrictions.
