# Supplementary material for: Analgesic and Functional Outcomes of the Pericapsular Nerve Group Block Versus the Quadratus Lumborum Block After Hip Surgery: A Systematic Review and Meta‐Analysis
Source: Pain Res Manag. 2026 Sep 6;2026:7392040. doi: 10.1155/prm/7392040 (PMC13547599; doi:10.1155/prm/7392040)
Supplement: Supplementary file 1 [file PRM-2026-7392040-s001.docx]

Comparative Efficacy of Pericapsular Nerve Group Block and Quadratus Lumborum Block for Postoperative Analgesia in Total Hip Arthroplasty: A Systematic Review and Meta-Analysis

Supplementary

Supplementary 1: Searching Strategies

Supplementary 2: Excluded Studies

Supplementary 3: Risk of Bias Diagram

Supplementary 4: Appraisal of the included studies, using the GRADE tool

Supplementary 5: Results of Meta-analysis

Supplementary 6: Adverse Events Reported by the Included Trials

Supplementary 7: Re-expression of the pooled pain effects on a common 0–10 scale and interpretation against the minimal clinically important difference (MCID).

Supplementary 1: Searching Strategies

Search Terms and Results:

| **Databases** | **Search Terms** | **Results** |
| --- | --- | --- |
| PubMed | ((Pericapsular nerve group[Title/Abstract] OR PENG[Title/Abstract]) OR (Quadratus lumborum[Title/Abstract] OR QLB[Title/Abstract] OR "QL block"[Title/Abstract])) AND (hip[Title/Abstract] OR "total hip arthroplasty"[Title/Abstract] OR THA[Title/Abstract] OR "hip fracture"[Title/Abstract] OR "hip joint"[Title/Abstract]) AND (Randomized Controlled Trial[Filter]) | 95 |
| Embase | (('pericapsular nerve group block':ti,ab,kw OR 'peng block':ti,ab,kw) OR ('quadratus lumborum block':ti,ab,kw OR 'qlb':ti,ab,kw OR 'ql block':ti,ab,kw)) AND ('hip surgery':ti,ab,kw OR 'total hip arthroplasty':ti,ab,kw OR 'tha':ti,ab,kw OR 'hip fracture':ti,ab,kw OR 'hip joint':ti,ab,kw) AND [randomized controlled trial]/lim | 333 |
| Web of Science | TS=(("pericapsular nerve group block" OR "peng block") OR ("quadratus lumborum block" OR "qlb" OR "ql block")) AND TS=("hip surgery" OR "total hip arthroplasty" OR "tha" OR "hip fracture" OR "hip joint") AND TS=("randomized controlled trial" OR "randomised controlled trial") | 56 |
| Cochrane Library | ("pericapsular nerve group block" OR "peng block" OR "quadratus lumborum block" OR "qlb" OR "ql block"):ti,ab,kw AND ("hip surgery" OR "total hip arthroplasty" OR "tha" OR "hip fracture" OR "hip joint"):ti,ab,kw | 644 |

Supplementary 2: Excluded Studies (total n=88)

| **No.** | **Author (year)** | **Title** | **Source** | **Reason for exclusion** |
| --- | --- | --- | --- | --- |
| 1 | Abdelfatah et al. (2024) | Effect of bupivacaine concentration on ultrasound-guided pericapsular group nerve block efficacy in hip surgery patients: comparative, randomized, double-blinded clinical trial | British Journal of Pain 18(5): 425–432. | PENG for spinal-positioning pain; comparator not QLB |
| 2 | Adel Abdelfattah Salama et al. (2025) | Comparison of Bupivacaine-Dexmedetomidine and Bupivacaine-Dexamethasone in PENG Block for Post-operative Analgesia in Hip Surgeries | Journal of Cellular and Molecular Anesthesia 10(3). | Comparator not QLB (adjuvant comparison within PENG) |
| 3 | Ahmed et al. (2024) | Efficacy of Pericapsular Nerve Group Block (PENG BLOCK) in the Perioperative Period for Patients Undergoing Hip Surgery | QJM: An International Journal of Medicine 117: ii41–ii42. | Comparator not QLB (PENG vs placebo/control) |
| 4 | Akgül et al. (2023) | COMPARISON OF LOCAL ANAESTHETIC CONCENTRATION IN PERICAPSULAR NERVE GROUP (PENG) BLOCK FOR TOTAL HIP ARTHROPLASTY: A PROSPECTIVE RANDOMIZED DOUBLE-BLIND CONTROLLED TRIAL | Regional Anesthesia and Pain Medicine 48: A26–A27. | Comparator not QLB (PENG vs placebo/control) |
| 5 | Alrefaey et al. (2020) | Pericapsular nerve group block for analgesia of positioning pain during spinal anesthesia in hip fracture patients, a randomized controlled study | Egyptian Journal of Anaesthesia 36(1): 234–239. | PENG for spinal-positioning pain; comparator not QLB |
| 6 | Aygun et al. (2022) | EVALUATION OF ULTRASOUND GUIDED PERICAPSULAR NERVE GROUP BLOCK ON NEURAXIAL ANESTHESIA POSITIONING PAIN, POSTOPERATIVE ANALGESIA AND QUALITY OF LIFE (QOR-15) IN PATIENTS UNDERGOING HIP SURGERY | Regional Anesthesia and Pain Medicine 47: A101–A102. | PENG for spinal-positioning pain; comparator not QLB |
| 7 | Aygun et al. (2023) | Effect of ultrasound-guided pericapsular nerve group (PENG) block on pain during patient positioning for central nervous blockade in hip surgery: a randomized controlled trial | BMC Anesthesiology 23(1). | PENG for spinal-positioning pain; comparator not QLB |
| 8 | Babu et al. (2025) | A RANDOMIZED CONTROLLED STUDY ON THE ANALGESIC EFFECTIVENESS OF PERICAPSULAR NERVE BLOCK COMPARED TO INTRAVENOUS FENTANYL FOR PATIENT POSITIONING DURING SPINAL ANESTHESIA IN PROXIMAL HIP FRACTURE SURGERIES | International Journal of Medicine and Public Health 15(2): 763–799. | PENG for spinal-positioning pain; comparator not QLB |
| 9 | Balakrishnan et al. (2023) | Comparison of continuous transmuscular quadratus lumborum block and continuous psoas compartment block for posterior total hip arthroplasty: A randomised controlled trial | Indian Journal of Anaesthesia 67(6): 530–536. | No PENG arm (QLB vs psoas compartment block) |
| 10 | Balakrishnan et al. (2025) | Perioperative Cardiac Biomarkers in Geriatric Hip Surgeries: A Comparison of Anterior IPB and PENG Blocks | Indian Heart Journal 77: S53–S54. | Comparator not QLB (PENG vs another regional technique) |
| 11 | Balasubramaniam et al. (2023) | Ultrasound-Guided Pericapsular Nerve Group Block for Hip Surgery: A Randomized Controlled Trial Study Comparing Ropivacaine and Ropivacaine With Dexamethasone | Cureus 15(1): e34261. | PENG for spinal-positioning pain; comparator not QLB |
| 12 | Bontozis et al. (2025) | INTRATHECAL NALBUPHINE WITH LEVOBUPIVACAINE FOR SPINAL ANESTHESIA IN TOTAL HIP ARTHROPLASTY: A PATH TO BETTER POSTOPERATIVE ANALGESIA | Pain Practice 25. | Comparator not QLB (adjuvant comparison within PENG) |
| 13 | Bontozis et al. (2025) | THE EFFECT OF NALBUPHINE AS AN ADJUVANT TO LEVOBUPIVACAINE IN SUBARACHNOID ANESTHESIA IN TOTAL HIP ARTHROPLASTY. A DOUBLE-BLIND, RANDOMIZED, CONTROLLED STUDY | Regional Anesthesia and Pain Medicine 50: A305–A306. | Comparator not QLB (adjuvant comparison within PENG) |
| 14 | Brixel et al. (2021) | Posterior Quadratus Lumborum Block in Total Hip Arthroplasty: A Randomized Controlled Trial | Anesthesiology 134(5): 722–733. | No PENG arm (QLB vs placebo/standard care) |
| 15 | Cakmak et al. (2025) | Comparison of Ultrasound-Guided Pericapsular Nerve Group Block Versus Intravenous Ketamine for Positional Pain Management Before Spinal Anesthesia in Patients Who Underwent Hip Fracture Surgery: A Randomized Controlled Trial | Medical science monitor : international medical journal of experimental and clinical research 31: e950517. | PENG for spinal-positioning pain; comparator not QLB |
| 16 | Calati et al. (2025) | Effectiveness of pericapsular nerve group block for hip fracture pain management in the emergency department: results of the ED-PENG-B randomised controlled trial | BMC Emergency Medicine 25(1). | Comparator not QLB (PENG vs systemic/neuraxial analgesia) |
| 17 | Chhabra et al. (2019) | Comparison of ultrasound guided continuous transmuscular quadratus lumborum block with ultrasound guided continuous psoas compartment block for total hip arthroplasty-a randomised controlled trial | Regional Anesthesia and Pain Medicine 44(10): A135. | No PENG arm (QLB vs psoas compartment block) |
| 18 | Chiang et al. (2023) | A Comparison of Pericapsular Nerve Group Block and Intravenous Morphine in Patients With Hip Fracture | Academic Emergency Medicine 30: 223. | Comparator not QLB (PENG vs systemic/neuraxial analgesia) |
| 19 | Cho et al. (2025) | Pericapsular Nerve Group Block with Periarticular Injection for Pain Management after Total Hip Arthroplasty: A Randomized Controlled Trial | Yonsei Medical Journal 66(4): 233–239. | Comparator not QLB (PENG vs another regional technique) |
| 20 | Chung et al. (2022) | Reduced Opioid Consumption with Pericapsular Nerve Group Block for Hip Surgery: A Randomized, Double-Blind, Placebo-Controlled Trial | Pain Research and Management 2022. | Comparator not QLB (PENG vs placebo/control) |
| 21 | Coppola et al. (2021) | Posterior Quadratus Lumborum Block in Total Hip Arthroplasty: A Randomized Controlled Trial | Anesthesiology 134(5): 774–783. | No PENG arm (QLB vs placebo/standard care) |
| 22 | Deri et al. (2024) | EFFICIENCY OF THE PERICAPSULAR NERVE GROUP (PENG) BLOCK ON POSTOPERATIVE ANALGESIA AND FUNCTIONAL RECOVERY FOLLOWING TOTAL HIP ARTHROPLASTY | Pain Practice 24: 31. | Comparator not QLB (PENG vs placebo/control) |
| 23 | Domagalska et al. (2023) | Pain Management and Functional Recovery after Pericapsular Nerve Group (PENG) Block for Total Hip Arthroplasty: A Prospective, Randomized, Double-Blinded Clinical Trial | Journal of Clinical Medicine 12(15). | Comparator not QLB (PENG vs placebo/control) |
| 24 | Eldeeb et al. (2024) | Effectiveness of Ultrasound Guided Quadratus Lumborum Block versus Intravenous Analgesia for Postoperative Pain Management in Patients Undergoing Hip Surgeries | QJM: An International Journal of Medicine 117: ii3. | No PENG arm (QLB vs placebo/standard care) |
| 25 | ElHalim et al. (2021) | Peng (pericapsular nerve group) block versus intravenous fentanyl as an analgesic technique in cardiac patients for positioning ofhip fracture: prospective, randomized study | Journal of Cardiovascular Disease Research 12(5): 565–568. | PENG for spinal-positioning pain; comparator not QLB |
| 26 | Erdem et al. (2026) | Effect of pericapsular nerve group block on postoperative cognitive function in older patients undergoing total hip arthroplasty | Die Anaesthesiologie. | Comparator not QLB (PENG vs placebo/control) |
| 27 | Ezhevskaya et al. (2024) | Ultrasound-assisted quadratus lumborum block as a method of postoperative analgesia in total hip arthroplasty: a single-center prospective randomized study | Regional Anesthesia and Acute Pain Management 18(4): 344–355. | No PENG arm (QLB vs placebo/standard care) |
| 28 | Ferré et al. (2024) | Pericapsular nerve group (PENG) block combined with local infiltration analgesia is not superior to local infiltration analgesia for the management of postoperative pain after primary elective total hip arthroplasty: a prospective, randomized, controlled, single-blind trial | Heliyon 10(13): e33766. | Comparator not QLB (PENG dose/volume/concentration comparison) |
| 29 | Gutierrez et al. (2021) | Quadratus lumborum block type 3 versus lumbar plexus block in hip replacement surgery: A randomized, prospective, non-inferiority study | Regional Anesthesia and Pain Medicine 46(2): 111–117. | No PENG arm (QLB compared with a non-PENG technique) |
| 30 | Hanauer et al. (2025) | The PENG Block in Elective Primary Anterior Total Hip Arthroplasty Is Associated with Reduced Length of Stay: A Multidisciplinary Prospective Randomized Double-Blinded Controlled Trial | Journal of Bone and Joint Surgery 107(8): 805–810. | Comparator not QLB (PENG vs placebo/control) |
| 31 | Haskins et al. (2021) | Anterior Quadratus Lumborum Block Does Not Provide Superior Pain Control after Hip Arthroscopy: A Double-blinded Randomized Controlled Trial | Anesthesiology 135(3): 433–441. | Wrong procedure (hip arthroscopy, not THA/fracture surgery) |
| 32 | Hawas et al. (2023) | Evaluation of Ultrasound- Guided Quadratus Lumborum Block for Postoperative Analgesia after Hip Surgery | QJM: An International Journal of Medicine 116: i9–i10. | No PENG arm (QLB compared with a non-PENG technique) |
| 33 | He et al. (2020) | Ultrasound-Guided Transmuscular Quadratus Lumborum Block Reduces Postoperative Pain Intensity in Patients Undergoing Total Hip Arthroplasty: A Randomized, Double-Blind, Placebo-Controlled Trial | Pain Research and Management 2020. | No PENG arm (QLB vs placebo/standard care) |
| 34 | He et al. (2018) | Effects and safety of quadratus lumborum block in analgesia after hip arthroplasty | National Medical Journal of China 98(8): 565–569. | No PENG arm (QLB vs placebo/standard care) |
| 35 | Hoda et al. (2025) | Ultrasound-Guided Pericapsular Nerve Group Block for Hip Surgery: a Randomized Controlled Trial Comparing Levobupivacaine Alone Versus Levobupivacaine With Dexamethasone | Cureus 17(5): e84568. | PENG for spinal-positioning pain; comparator not QLB |
| 36 | Hu et al. (2024) | Minimum effective concentration of ropivacaine for ultrasound-guided transmuscular quadratus lumborum block in total hip arthroplasty: a randomized clinical trial | Brazilian Journal of Anesthesiology (English Edition) 74(2). | No PENG arm (QLB compared with a non-PENG technique) |
| 37 | Hu et al. (2025) | A modified hip pericapsular nerve block on postoperative pain and functional outcome after total hip arthroplasty: a prospective, double-blind, randomized controlled study | Korean J Anesthesiol 78(4): 351–360. | Comparator not QLB (PENG vs placebo/control) |
| 38 | Hu et al. (2023) | Efficacy of Ultrasound-Guided Pericapsular Nerve Group (PENG) Block Combined With Local Infiltration Analgesia on Postoperative Pain After Total Hip Arthroplasty: A Prospective, Double-Blind, Randomized Controlled Trial | Journal of Arthroplasty 38(6): 1096–1103. | Comparator not QLB (PENG vs placebo/control) |
| 39 | Huang et al. (2021) | Postoperative analgesic effects of the quadratus lumborum block III and transversalis fascia plane block in paediatric patients with developmental dysplasia of the hip undergoing open reduction surgeries: a double-blinded randomised controlled trial | BMJ Open 11(2): e038992. | Pediatric population |
| 40 | Huang et al. (2024) | Effect of Pericapsular Nerve Group Block with Different Concentrations and Volumes of Ropivacaine on Functional Recovery in Total Hip Arthroplasty: a Randomized, Observer-Masked, Controlled Trial | Journal of Pain Research 17: 2639–2640. | Comparator not QLB (PENG dose/volume/concentration comparison) |
| 41 | Iglesias et al. (2023) | [Translated article] Pericapsular nerves block (PENG) is an effective and safe alternative for postoperative pain management after primary total hip arthroplasty: a randomised clinical trial | Revista espanola de cirugia ortopedica y traumatologia 67(3): T226–T232. | Comparator not QLB (PENG vs systemic/neuraxial analgesia) |
| 42 | Kamal et al. (2025) | Evaluation of the Efficacy of 0.2% Ropivacaine Combined with Dexamethasone versus Fentanyl in Pericapsular Nerve Group (PENG) Block for Postoperative Analgesia in Hip Surgery Patients | International Journal of Pharmaceutical Quality Assurance 16(2): 321–325. | Comparator not QLB (adjuvant comparison within PENG) |
| 43 | Kelly et al. (2022) | Comparison of lateral quadratus lumborum and lumbar plexus blocks for postoperative analgesia following total hip arthroplasty: A randomized clinical trial | Regional Anesthesia and Pain Medicine 47(9): 541–546. | No PENG arm (QLB compared with a non-PENG technique) |
| 44 | Kikuchi et al. (2021) | Anterior quadratus lumborum block for postoperative recovery after total hip arthroplasty: A randomized, multiple-blind, placebo-controlled trial | Regional Anesthesia and Pain Medicine 70(SUPPL 1): A3. | No PENG arm (QLB vs placebo/standard care) |
| 45 | Kim et al. (2025) | Iliopsoas plane block does not improve pain after primary total hip arthroplasty in the presence of multimodal analgesia: a single institution randomized controlled trial | Regional Anesthesia and Pain Medicine 50(3): 257–263. | Did not meet PICOS (no PENG/QLB comparison) |
| 46 | Küçük et al. (2024) | Comparison of the Analgesic Effect of Pericapsular Nerve Group Block and Lumbar Erector Spinae Plane Block in Elective Hip Surgery | Medicina (Kaunas, Lithuania) 60(5). | Comparator not QLB (PENG vs placebo/control) |
| 47 | Kukreja et al. (2019) | Anterior quadratus lumborum block analgesia for total hip arthroplasty: A randomized, controlled study | Regional Anesthesia and Pain Medicine 44(12): 1075–1079. | No PENG arm (QLB vs placebo/standard care) |
| 48 | Kukreja et al. (2023) | Quality of recovery after pericapsular nerve group (PENG) block for primary total hip arthroplasty under spinal anaesthesia: a randomised controlled observer-blinded trial | British Journal of Anaesthesia 130(6): 773–779. | Comparator not QLB (PENG vs placebo/control) |
| 49 | Lee et al. (2024) | A Randomized Controlled Trial to Evaluate the Analgesic Effectiveness of Periarticular Injections and Pericapsular Nerve Group Block for Patients Undergoing Total Hip Arthroplasty | Journal of Personalized Medicine 14(4). | Comparator not QLB (PENG vs another regional technique) |
| 50 | Lee et al. (2024) | Comparing the Pericapsular Nerve Group Block and the Lumbar Plexus Block for Hip Fracture Surgery: A Single-Center Randomized Double-Blinded Study | Journal of Clinical Medicine 13(1). | Comparator not QLB (PENG vs another regional technique) |
| 51 | Lin et al. (2022) | The Pericapsular Nerve Group (PENG) block combined with Local Infiltration Analgesia (LIA) compared to placebo and LIA in hip arthroplasty surgery: a multi-center double-blinded randomized-controlled trial | BMC Anesthesiology 22(1). | Comparator not QLB (PENG vs placebo/control) |
| 52 | Lin et al. (2023) | Pericapsular nerve group (PENG) block for early pain management of elderly patients with hip fracture: a single-center double-blind randomized controlled trial | Regional Anesthesia and Pain Medicine 48(11): 535–539. | Comparator not QLB (PENG vs placebo/control) |
| 53 | Mafizer et al. (2026) | Impact of bupivacaine dose on haemodynamics in elderly hip surgery: a randomized controlled trial | BMC geriatrics. | Did not meet PICOS (no PENG/QLB comparison) |
| 54 | Manuwong et al. (2019) | Efficacy of transmuscular quadratus lumborum (Ql-3) block for analgesia after total hip arthroplasty: A randomized controlled trial | Regional Anesthesia and Pain Medicine 44(10): A118. | No PENG arm (QLB vs placebo/standard care) |
| 55 | Medhat et al. (2023) | The Analgesic Effects of Preemptive Ultrasound-Guided Pericapsular Nerve Group Block in Comparison with Erector Spinae Plane Block in Elderly Undergoing Hip Arthroplasty: a Randomized Controlled Trial | Anesthesiology and pain medicine 13(5): e138623. | PENG for spinal-positioning pain; comparator not QLB |
| 56 | Nambiar et al. (2024) | Comparison of analgesic efficacy of parasacral sciatic and pericapsular nerve block versus pericapsular nerve block in patients undergoing total hip arthroplasty: a randomised controlled trial | Regional Anesthesia and Pain Medicine 49: A107. | Comparator not QLB (PENG vs systemic/neuraxial analgesia) |
| 57 | Nana et al. (2022) | Ultrasound-Guided Oblique Sagittal Anterior Quadratus Lumborum Block in Total Hip Arthroplasty: a Randomized Controlled Trial | Pain Physician 25(4): E609. | No PENG arm (QLB compared with a non-PENG technique) |
| 58 | Neji et al. (2023) | EFFICACY OF PERICAPSULAR NERVE GROUP BLOCK AFTER TOTAL HIP ARTHROPLASTY SURGERY | Regional Anesthesia and Pain Medicine 48: A120–A121. | PENG for spinal-positioning pain; comparator not QLB |
| 59 | Oremuš et al. (2025) | Pericapsular nerve group (PENG) block compared to intrathecal morphine for analgesic efficacy in total hip arthroplasty: A placebo-controlled randomized double-blind non-inferiority trial | Journal of Clinical Anesthesia 106. | Comparator not QLB (PENG vs placebo/control) |
| 60 | Özgüner et al. (2025) | Different local anesthetic volumes in pericapsular nerve group block for hip fracture patients | Turkish Journal of Medical Sciences 55(4): 860–867. | Comparator not QLB (PENG dose/volume/concentration comparison) |
| 61 | Pascarella et al. (2021) | Impact of the pericapsular nerve group (PENG) block on postoperative analgesia and functional recovery following total hip arthroplasty: a randomised, observer-masked, controlled trial | Anaesthesia 76(11): 1492–1498. | Comparator not QLB (PENG vs placebo/control) |
| 62 | Paturi et al. (2025) | A RANDOMIZED COMPARATIVE STUDY OF CLONIDINE AS AN ADJUVANT TO ROPIVACAINE IN ULTRASOUND GUIDED PERICAPSULAR NERVE GROUP (PENG) BLOCK FOR POST OPERATIVE ANALGESIA IN HIP SURGERIES | International Journal of Academic Medicine and Pharmacy 7(4): 803–806. | Comparator not QLB (adjuvant comparison within PENG) |
| 63 | Peng et al. (2025) | Preoperative Analgesia Efficacy of Liposomal Bupivacaine Following Pericapsular Nerve Group (PENG) Block in Patients with Hip Fracture: a Randomized Controlled Observer-Blinded Study | Pain and Therapy 14(1): 283–296. | Comparator not QLB (PENG vs systemic/neuraxial analgesia) |
| 64 | Polania Gutierrez et al. (2021) | Quadratus lumborum block type 3 versus lumbar plexus block in hip replacement surgery: a randomized, prospective, non-inferiority study | Reg Anesth Pain Med 46(2): 111–117. | No PENG arm (QLB compared with a non-PENG technique) |
| 65 | Rey et al. (2024) | PENG block associated with intraarticular block for perioperative analgesia in hip surgery | Regional Anesthesia and Pain Medicine 49: A292. | PENG for spinal-positioning pain; comparator not QLB |
| 66 | Reysner et al. (2025) | Chemical ablation of pericapsular nerve group with 95% ethanol for pain relief and quality of life in patients with hip osteoarthritis: a prospective, double-blinded, randomised, controlled trial | Br J Anaesth 135(2): 382–389. | Comparator not QLB (PENG vs placebo/control) |
| 67 | Reysner et al. (2025) | PERINEURAL DEXAMETHASONE FOR PENG BLOCK IN GERIATRIC PATIENTS UNDERGOING TOTAL HIP ARTHROPLASTY: A RANDOMIZED, DOUBLE-BLINDED CLINICAL TRIAL | Regional Anesthesia and Pain Medicine 50: A2–A3. | Comparator not QLB (adjuvant comparison within PENG) |
| 68 | Reysner et al. (2025) | Perineural dexamethasone effectively prolongs anaesthesic block duration in total hip arthroplasty, reduces opioid consumption, and does not compromise motor function, nerve integrity, or glycaemic control | International Orthopaedics 49(8): 1791–1799. | Comparator not QLB (adjuvant comparison within PENG) |
| 69 | Reysner et al. (2025) | Erector spinae plane block (ESPB) vs. pericapsular nerve group (PENG) block in total hip arthroplasty in elderly patients: a randomized, double-blinded, controlled trial | Anaesthesiology Intensive Therapy 57(1): e90–e98. | Comparator not QLB (PENG vs placebo/control) |
| 70 | Reysner et al. (2025) | Reduced Ropivacaine Volume with Perineural Dexamethasone in PENG Block for Total Hip Arthroplasty: A Randomized Controlled Trial | Journal of Clinical Medicine 14(24). | Comparator not QLB (adjuvant comparison within PENG) |
| 71 | Rozier et al. (2025) | Anterior quadratus lumborum block in total hip arthroplasty: A two-center, randomized, placebo-controlled trial showing no additional benefit over multimodal analgesia | Regional Anesthesia and Pain Medicine. | No PENG arm (QLB vs placebo/standard care) |
| 72 | Salgado-García et al. (2024) | Comparison of the Analgesic Efficacy between Levobupivacaine 0.25% and Ropivacaine 0.375% for PENG (Pericapsular Nerve Group) Block in the Context of Hip Fracture Surgery of Elderly Patients: A Single-Center, Randomized, and Controlled Clinical Trial | Journal of Clinical Medicine 13(3). | Comparator not QLB (PENG dose/volume/concentration comparison) |
| 73 | Sharma et al. (2024) | Evaluation of two doses (20 mL and 15 mL) of 0.25% bupivacaine in pericapsular nerve group block for patient positioning for sub-arachnoid block during hip fracture surgery: A single-centre, randomised comparative trial | Indian Journal of Anaesthesia 68(4): 323–328. | PENG for spinal-positioning pain; comparator not QLB |
| 74 | Sonmez et al. (2024) | The effect of pericapsular nerve group block on perioperative opioid usage in femur fracture cases monitored with nociceptive level monitoring device | Regional Anesthesia and Pain Medicine 49: A116. | Comparator not QLB (PENG vs placebo/control) |
| 75 | Turkan et al. (2025) | Effects of pericapsular nerve group block on patients under spinal anesthesia for total hip arthroplasty: a randomized, controlled, double-blind study | Signa Vitae 21(4): 70–76. | Comparator not QLB (PENG vs placebo/control) |
| 76 | Umeh et al. (2023) | Transmuscular Quadratus Lumborum Block Does Not Provide Significant Benefit for Primary Hip Arthroscopy with Pericapsular Infiltration: A Randomized Control Trial | Arthroscopy 39(12): 2456–2463. | Wrong procedure (hip arthroscopy, not THA/fracture surgery) |
| 77 | Umin (2018) | The efficacy of the Quadratus Lumborum Block anterior approach for the postoperative recovery after Total Hip Arthroplasty. A single-center, double-blinded, randomized controlled trial | https://trialsearch.who.int/Trial2.aspx?TrialID=JPRN-UMIN000032255. | No PENG arm (QLB vs placebo/standard care) |
| 78 | Vijayan et al. (2026) | Efficacy of Ultrasound-Guided PENG Block for Acute Pain Relief in Adult Hip Fracture Patients: A Double-Blind Randomized Trial | Academic Emergency Medicine 33(3). | Comparator not QLB (PENG vs placebo/control) |
| 79 | Villa et al. (2025) | PERICAPSULAR NERVE GROUP BLOCK VS LUMBAR PLEXUS FOR HIP SURGERY: A RANDOMIZED DOUBLEBLIND TRIAL. PRELIMINARY RESULTS | Regional Anesthesia and Pain Medicine 50: A262–A263. | Comparator not QLB (PENG vs placebo/control) |
| 80 | Wang et al. (2022) | Ultrasound-Guided Oblique Sagittal Anterior Quadratus Lumborum Block in Total Hip Arthroplasty: A Randomized Controlled Trial | Pain Physician 25(4): E609–E617. | No PENG arm (QLB vs placebo/standard care) |
| 81 | Wang et al. (2024) | Effect of Pericapsular Nerve Group Block with Different Concentrations and Volumes of Ropivacaine on Functional Recovery in Total Hip Arthroplasty: a Randomized, Observer-Masked, Controlled Trial | Journal of Pain Research 17: 2345–2346. | Comparator not QLB (PENG dose/volume/concentration comparison) |
| 82 | Wen et al. (2024) | Effects of Different Volumes of Ropivacaine for Pericapsular Nerve Group Block on Incidence of Quadriceps Weakness and Analgesic Efficacy Following Hip Arthroplasty: A Randomized Controlled Trial | Pain and Therapy 13(3): 533–541. | Comparator not QLB (PENG dose/volume/concentration comparison) |
| 83 | Wilson et al. (2022) | Preoperative Quadratus Lumborum Block Reduces Opioid Requirements in the Immediate Postoperative Period Following Hip Arthroscopy: A Randomized, Blinded Clinical Trial | Arthroscopy 38(3): 808–815. | Wrong procedure (hip arthroscopy, not THA/fracture surgery) |
| 84 | Yuan et al. (2022) | Comparative efficacy analysis of ultrasound-guided quadratus lumborum block and lumbar plexus block in hip arthroscopy: a pilot prospective randomized controlled trial | Journal of Hip Preservation Surgery 9(2): 119–125. | Wrong procedure (hip arthroscopy, not THA/fracture surgery) |
| 85 | Yuanfeng et al. (2025) | Dose-effect relationship between dexmedetomidine and ropivacaine during pericapsular nerve group block in elderly patients undergoing total hip arthroplasty | Chinese Journal of Tissue Engineering Research 29(21): 4514–4520. | Comparator not QLB (PENG vs placebo/control) |
| 86 | Zheng et al. (2022) | Preoperative pericapsular nerve group (PENG) block for total hip arthroplasty: A randomized, placebo-controlled trial | Regional Anesthesia and Pain Medicine 47(3): 155–160. | Comparator not QLB (PENG vs placebo/control) |
| 87 | Zhu et al. (2023) | Effects of anterior approach to quadratus lumborum block on postoperative cognitive function following hip surgery in older people: a randomized controlled clinical trial | BMC geriatrics 23(1): 835. | No PENG arm (QLB vs placebo/standard care) |
| 88 | Zhu et al. (2024) | Effects of pericapsular nerve group block on early postoperative cognitive function in older people undergoing hip arthroplasty: a randomized controlled clinical trial | BMC Geriatr 24(1): 931. | Comparator not QLB (PENG vs placebo/control) |

Supplementary 3: Risk of Bias Diagram

| **Unique ID** | Abdelsalam 2024 | **Assessor** | YLT |
| --- | --- | --- | --- |
| **Domain** | **Signalling question** | **Response** | **Comments** |
| **Bias arising from the randomization process** | 1.1 Was the allocation sequence random? | Y |  |
|  | 1.2 Was the allocation sequence concealed until participants were enrolled and assigned to interventions? | Y |  |
|  | 1.3 Did baseline differences between intervention groups suggest a problem with the randomization process? | N |  |
|  | **Risk of bias judgement** | **Low** |  |
| **Bias due to deviations from intended interventions** | 2.1.Were participants aware of their assigned intervention during the trial? | N |  |
|  | 2.2.Were carers and people delivering the interventions aware of participants' assigned intervention during the trial? | N |  |
|  | 2.3. If Y/PY/NI to 2.1 or 2.2: Were there deviations from the intended intervention that arose because of the experimental context? | NA |  |
|  | 2.4 If Y/PY to 2.3: Were these deviations likely to have affected the outcome? | NA |  |
|  | 2.5. If Y/PY/NI to 2.4: Were these deviations from intended intervention balanced between groups? | NA |  |
|  | 2.6 Was an appropriate analysis used to estimate the effect of assignment to intervention? | PY |  |
|  | 2.7 If N/PN/NI to 2.6: Was there potential for a substantial impact (on the result) of the failure to analyse participants in the group to which they were randomized? | NA |  |
|  | **Risk of bias judgement** | **Low** |  |
| **Bias due to missing outcome data** | 3.1 Were data for this outcome available for all, or nearly all, participants randomized? | PY |  |
|  | 3.2 If N/PN/NI to 3.1: Is there evidence that result was not biased by missing outcome data? | NA |  |
|  | 3.3 If N/PN to 3.2: Could missingness in the outcome depend on its true value? | NA |  |
|  | 3.4 If Y/PY/NI to 3.3: Is it likely that missingness in the outcome depended on its true value? | NA |  |
|  | **Risk of bias judgement** | **Low** |  |
| **Bias in measurement of the outcome** | 4.1 Was the method of measuring the outcome inappropriate? | PN |  |
|  | 4.2 Could measurement or ascertainment of the outcome have differed between intervention groups? | PN |  |
|  | 4.3 Were outcome assessors aware of the intervention received by study participants? | N |  |
|  | 4.4 If Y/PY/NI to 4.3: Could assessment of the outcome have been influenced by knowledge of intervention received? | NA |  |
|  | 4.5 If Y/PY/NI to 4.4: Is it likely that assessment of the outcome was influenced by knowledge of intervention received? | NA |  |
|  | **Risk of bias judgement** | **Low** |  |
| **Bias in selection of the reported result** | 5.1 Were the data that produced this result analysed in accordance with a pre-specified analysis plan that was finalized before unblinded outcome data were available for analysis? | PN |  |
|  | 5.2 ... multiple eligible outcome measurements (e.g. scales, definitions, time points) within the outcome domain? | PY |  |
|  | 5.3 ... multiple eligible analyses of the data? | PN |  |
|  | **Risk of bias judgement** | **Some concerns** |  |
| **Overall bias** | **Risk of bias judgement** | **Some concerns** |  |

| **Unique ID** | Aslan 2025 | **Assessor** | INH |
| --- | --- | --- | --- |
| **Domain** | **Signalling question** | **Response** | **Comments** |
| **Bias arising from the randomization process** | 1.1 Was the allocation sequence random? | Y |  |
|  | 1.2 Was the allocation sequence concealed until participants were enrolled and assigned to interventions? | Y |  |
|  | 1.3 Did baseline differences between intervention groups suggest a problem with the randomization process? | PN |  |
|  | **Risk of bias judgement** | **Low** |  |
| **Bias due to deviations from intended interventions** | 2.1.Were participants aware of their assigned intervention during the trial? | PY |  |
|  | 2.2.Were carers and people delivering the interventions aware of participants' assigned intervention during the trial? | PY |  |
|  | 2.3. If Y/PY/NI to 2.1 or 2.2: Were there deviations from the intended intervention that arose because of the experimental context? | PN |  |
|  | 2.4 If Y/PY to 2.3: Were these deviations likely to have affected the outcome? | NA |  |
|  | 2.5. If Y/PY/NI to 2.4: Were these deviations from intended intervention balanced between groups? | NA |  |
|  | 2.6 Was an appropriate analysis used to estimate the effect of assignment to intervention? | PY |  |
|  | 2.7 If N/PN/NI to 2.6: Was there potential for a substantial impact (on the result) of the failure to analyse participants in the group to which they were randomized? | NA |  |
|  | **Risk of bias judgement** | **Low** |  |
| **Bias due to missing outcome data** | 3.1 Were data for this outcome available for all, or nearly all, participants randomized? | PY |  |
|  | 3.2 If N/PN/NI to 3.1: Is there evidence that result was not biased by missing outcome data? | NA |  |
|  | 3.3 If N/PN to 3.2: Could missingness in the outcome depend on its true value? | NA |  |
|  | 3.4 If Y/PY/NI to 3.3: Is it likely that missingness in the outcome depended on its true value? | NA |  |
|  | **Risk of bias judgement** | **Low** |  |
| **Bias in measurement of the outcome** | 4.1 Was the method of measuring the outcome inappropriate? | PN |  |
|  | 4.2 Could measurement or ascertainment of the outcome have differed between intervention groups? | N |  |
|  | 4.3 Were outcome assessors aware of the intervention received by study participants? | PY |  |
|  | 4.4 If Y/PY/NI to 4.3: Could assessment of the outcome have been influenced by knowledge of intervention received? | PN |  |
|  | 4.5 If Y/PY/NI to 4.4: Is it likely that assessment of the outcome was influenced by knowledge of intervention received? | NA |  |
|  | **Risk of bias judgement** | **Low** |  |
| **Bias in selection of the reported result** | 5.1 Were the data that produced this result analysed in accordance with a pre-specified analysis plan that was finalized before unblinded outcome data were available for analysis? | PY |  |
|  | 5.2 ... multiple eligible outcome measurements (e.g. scales, definitions, time points) within the outcome domain? | PN |  |
|  | 5.3 ... multiple eligible analyses of the data? | PN |  |
|  | **Risk of bias judgement** | **Low** |  |
| **Overall bias** | **Risk of bias judgement** | **Low** |  |

| **Unique ID** | Elshall 2025 | **Assessor** | INH |
| --- | --- | --- | --- |
| **Domain** | Signalling question | **Response** | Comments |
| **Bias arising from the randomization process** | 1.1 Was the allocation sequence random? | **Y** |  |
|  | 1.2 Was the allocation sequence concealed until participants were enrolled and assigned to interventions? | **Y** |  |
|  | 1.3 Did baseline differences between intervention groups suggest a problem with the randomization process? | **N** |  |
|  | Risk of bias judgement | **Low** |  |
| **Bias due to deviations from intended interventions** | 2.1.Were participants aware of their assigned intervention during the trial? | **N** |  |
|  | 2.2.Were carers and people delivering the interventions aware of participants' assigned intervention during the trial? | **N** |  |
|  | 2.3. If Y/PY/NI to 2.1 or 2.2: Were there deviations from the intended intervention that arose because of the experimental context? | **NA** |  |
|  | 2.4 If Y/PY to 2.3: Were these deviations likely to have affected the outcome? | **NA** |  |
|  | 2.5. If Y/PY/NI to 2.4: Were these deviations from intended intervention balanced between groups? | **NA** |  |
|  | 2.6 Was an appropriate analysis used to estimate the effect of assignment to intervention? | **Y** |  |
|  | 2.7 If N/PN/NI to 2.6: Was there potential for a substantial impact (on the result) of the failure to analyse participants in the group to which they were randomized? | **NA** |  |
|  | Risk of bias judgement | **Low** |  |
| **Bias due to missing outcome data** | 3.1 Were data for this outcome available for all, or nearly all, participants randomized? | **Y** |  |
|  | 3.2 If N/PN/NI to 3.1: Is there evidence that result was not biased by missing outcome data? | **NA** |  |
|  | 3.3 If N/PN to 3.2: Could missingness in the outcome depend on its true value? | **NA** |  |
|  | 3.4 If Y/PY/NI to 3.3: Is it likely that missingness in the outcome depended on its true value? | **NA** |  |
|  | Risk of bias judgement | **Low** |  |
| **Bias in measurement of the outcome** | 4.1 Was the method of measuring the outcome inappropriate? | **N** |  |
|  | 4.2 Could measurement or ascertainment of the outcome have differed between intervention groups? | **N** |  |
|  | 4.3 Were outcome assessors aware of the intervention received by study participants? | **N** |  |
|  | 4.4 If Y/PY/NI to 4.3: Could assessment of the outcome have been influenced by knowledge of intervention received? | **NA** |  |
|  | 4.5 If Y/PY/NI to 4.4: Is it likely that assessment of the outcome was influenced by knowledge of intervention received? | **NA** |  |
|  | Risk of bias judgement | **Low** |  |
| **Bias in selection of the reported result** | 5.1 Were the data that produced this result analysed in accordance with a pre-specified analysis plan that was finalized before unblinded outcome data were available for analysis? | **Y** |  |
|  | 5.2 ... multiple eligible outcome measurements (e.g. scales, definitions, time points) within the outcome domain? | **PN** |  |
|  | 5.3 ... multiple eligible analyses of the data? | **PN** |  |
|  | Risk of bias judgement | **Low** |  |
| **Overall bias** | Risk of bias judgement | **Low** |  |

| **Unique ID** | Et 2023 | **Assessor** | INH |
| --- | --- | --- | --- |
| **Domain** | **Signalling question** | **Response** | **Comments** |
| **Bias arising from the randomization process** | 1.1 Was the allocation sequence random? | Y |  |
|  | 1.2 Was the allocation sequence concealed until participants were enrolled and assigned to interventions? | Y |  |
|  | 1.3 Did baseline differences between intervention groups suggest a problem with the randomization process? | PN |  |
|  | **Risk of bias judgement** | **Low** |  |
| **Bias due to deviations from intended interventions** | 2.1.Were participants aware of their assigned intervention during the trial? | N |  |
|  | 2.2.Were carers and people delivering the interventions aware of participants' assigned intervention during the trial? | N |  |
|  | 2.3. If Y/PY/NI to 2.1 or 2.2: Were there deviations from the intended intervention that arose because of the experimental context? | NA |  |
|  | 2.4 If Y/PY to 2.3: Were these deviations likely to have affected the outcome? | NA |  |
|  | 2.5. If Y/PY/NI to 2.4: Were these deviations from intended intervention balanced between groups? | NA |  |
|  | 2.6 Was an appropriate analysis used to estimate the effect of assignment to intervention? | Y |  |
|  | 2.7 If N/PN/NI to 2.6: Was there potential for a substantial impact (on the result) of the failure to analyse participants in the group to which they were randomized? | NA |  |
|  | **Risk of bias judgement** | **Low** |  |
| **Bias due to missing outcome data** | 3.1 Were data for this outcome available for all, or nearly all, participants randomized? | Y |  |
|  | 3.2 If N/PN/NI to 3.1: Is there evidence that result was not biased by missing outcome data? | NA |  |
|  | 3.3 If N/PN to 3.2: Could missingness in the outcome depend on its true value? | NA |  |
|  | 3.4 If Y/PY/NI to 3.3: Is it likely that missingness in the outcome depended on its true value? | NA |  |
|  | **Risk of bias judgement** | **Low** |  |
| **Bias in measurement of the outcome** | 4.1 Was the method of measuring the outcome inappropriate? | N |  |
|  | 4.2 Could measurement or ascertainment of the outcome have differed between intervention groups? | PN |  |
|  | 4.3 Were outcome assessors aware of the intervention received by study participants? | N |  |
|  | 4.4 If Y/PY/NI to 4.3: Could assessment of the outcome have been influenced by knowledge of intervention received? | NA |  |
|  | 4.5 If Y/PY/NI to 4.4: Is it likely that assessment of the outcome was influenced by knowledge of intervention received? | NA |  |
|  | **Risk of bias judgement** | **Low** |  |
| **Bias in selection of the reported result** | 5.1 Were the data that produced this result analysed in accordance with a pre-specified analysis plan that was finalized before unblinded outcome data were available for analysis? | Y |  |
|  | 5.2 ... multiple eligible outcome measurements (e.g. scales, definitions, time points) within the outcome domain? | PN |  |
|  | 5.3 ... multiple eligible analyses of the data? | PN |  |
|  | **Risk of bias judgement** | **Low** |  |
| **Overall bias** | **Risk of bias judgement** | **Low** |  |

| **Unique ID** | Hay 2026 | **Assessor** | INH |
| --- | --- | --- | --- |
| **Domain** | **Signalling question** | **Response** | **Comments** |
| **Bias arising from the randomization process** | 1.1 Was the allocation sequence random? | Y |  |
|  | 1.2 Was the allocation sequence concealed until participants were enrolled and assigned to interventions? | Y |  |
|  | 1.3 Did baseline differences between intervention groups suggest a problem with the randomization process? | N |  |
|  | **Risk of bias judgement** | **Low** |  |
| **Bias due to deviations from intended interventions** | 2.1.Were participants aware of their assigned intervention during the trial? | PY |  |
|  | 2.2.Were carers and people delivering the interventions aware of participants' assigned intervention during the trial? | PY |  |
|  | 2.3. If Y/PY/NI to 2.1 or 2.2: Were there deviations from the intended intervention that arose because of the experimental context? | PN |  |
|  | 2.4 If Y/PY to 2.3: Were these deviations likely to have affected the outcome? | NA |  |
|  | 2.5. If Y/PY/NI to 2.4: Were these deviations from intended intervention balanced between groups? | NA |  |
|  | 2.6 Was an appropriate analysis used to estimate the effect of assignment to intervention? | Y |  |
|  | 2.7 If N/PN/NI to 2.6: Was there potential for a substantial impact (on the result) of the failure to analyse participants in the group to which they were randomized? | NA |  |
|  | **Risk of bias judgement** | **Low** |  |
| **Bias due to missing outcome data** | 3.1 Were data for this outcome available for all, or nearly all, participants randomized? | PY |  |
|  | 3.2 If N/PN/NI to 3.1: Is there evidence that result was not biased by missing outcome data? | NA |  |
|  | 3.3 If N/PN to 3.2: Could missingness in the outcome depend on its true value? | NA |  |
|  | 3.4 If Y/PY/NI to 3.3: Is it likely that missingness in the outcome depended on its true value? | NA |  |
|  | **Risk of bias judgement** | **Low** |  |
| **Bias in measurement of the outcome** | 4.1 Was the method of measuring the outcome inappropriate? | PN |  |
|  | 4.2 Could measurement or ascertainment of the outcome have differed between intervention groups? | PN |  |
|  | 4.3 Were outcome assessors aware of the intervention received by study participants? | PN |  |
|  | 4.4 If Y/PY/NI to 4.3: Could assessment of the outcome have been influenced by knowledge of intervention received? | NA |  |
|  | 4.5 If Y/PY/NI to 4.4: Is it likely that assessment of the outcome was influenced by knowledge of intervention received? | NA |  |
|  | **Risk of bias judgement** | **Low** |  |
| **Bias in selection of the reported result** | 5.1 Were the data that produced this result analysed in accordance with a pre-specified analysis plan that was finalized before unblinded outcome data were available for analysis? | N |  |
|  | 5.2 ... multiple eligible outcome measurements (e.g. scales, definitions, time points) within the outcome domain? | N |  |
|  | 5.3 ... multiple eligible analyses of the data? | N |  |
|  | **Risk of bias judgement** | **Some concerns** |  |
| **Overall bias** | **Risk of bias judgement** | **Some concerns** |  |

| **Unique ID** | Şehirlioğlu 2025 | **Assessor** | YLT |
| --- | --- | --- | --- |
| **Domain** | **Signalling question** | **Response** | **Comments** |
| **Bias arising from the randomization process** | 1.1 Was the allocation sequence random? | Y |  |
|  | 1.2 Was the allocation sequence concealed until participants were enrolled and assigned to interventions? | Y |  |
|  | 1.3 Did baseline differences between intervention groups suggest a problem with the randomization process? | PY |  |
|  | **Risk of bias judgement** | **Some concerns** |  |
| **Bias due to deviations from intended interventions** | 2.1.Were participants aware of their assigned intervention during the trial? | N |  |
|  | 2.2.Were carers and people delivering the interventions aware of participants' assigned intervention during the trial? | N |  |
|  | 2.3. If Y/PY/NI to 2.1 or 2.2: Were there deviations from the intended intervention that arose because of the experimental context? | NA |  |
|  | 2.4 If Y/PY to 2.3: Were these deviations likely to have affected the outcome? | NA |  |
|  | 2.5. If Y/PY/NI to 2.4: Were these deviations from intended intervention balanced between groups? | NA |  |
|  | 2.6 Was an appropriate analysis used to estimate the effect of assignment to intervention? | Y |  |
|  | 2.7 If N/PN/NI to 2.6: Was there potential for a substantial impact (on the result) of the failure to analyse participants in the group to which they were randomized? | NA |  |
|  | **Risk of bias judgement** | **Low** |  |
| **Bias due to missing outcome data** | 3.1 Were data for this outcome available for all, or nearly all, participants randomized? | Y |  |
|  | 3.2 If N/PN/NI to 3.1: Is there evidence that result was not biased by missing outcome data? | NA |  |
|  | 3.3 If N/PN to 3.2: Could missingness in the outcome depend on its true value? | NA |  |
|  | 3.4 If Y/PY/NI to 3.3: Is it likely that missingness in the outcome depended on its true value? | NA |  |
|  | **Risk of bias judgement** | **Low** |  |
| **Bias in measurement of the outcome** | 4.1 Was the method of measuring the outcome inappropriate? | PN |  |
|  | 4.2 Could measurement or ascertainment of the outcome have differed between intervention groups? | PN |  |
|  | 4.3 Were outcome assessors aware of the intervention received by study participants? | N |  |
|  | 4.4 If Y/PY/NI to 4.3: Could assessment of the outcome have been influenced by knowledge of intervention received? | NA |  |
|  | 4.5 If Y/PY/NI to 4.4: Is it likely that assessment of the outcome was influenced by knowledge of intervention received? | NA |  |
|  | **Risk of bias judgement** | **Low** |  |
| **Bias in selection of the reported result** | 5.1 Were the data that produced this result analysed in accordance with a pre-specified analysis plan that was finalized before unblinded outcome data were available for analysis? | Y |  |
|  | 5.2 ... multiple eligible outcome measurements (e.g. scales, definitions, time points) within the outcome domain? | PN |  |
|  | 5.3 ... multiple eligible analyses of the data? | PN |  |
|  | **Risk of bias judgement** | **Low** |  |
| **Overall bias** | **Risk of bias judgement** | **Some concerns** |  |

| **Unique ID** | Wang 2023 | **Assessor** | YLT |
| --- | --- | --- | --- |
| **Domain** | **Signalling question** | **Response** | **Comments** |
| **Bias arising from the randomization process** | 1.1 Was the allocation sequence random? | Y |  |
|  | 1.2 Was the allocation sequence concealed until participants were enrolled and assigned to interventions? | Y |  |
|  | 1.3 Did baseline differences between intervention groups suggest a problem with the randomization process? | PN |  |
|  | **Risk of bias judgement** | **Low** |  |
| **Bias due to deviations from intended interventions** | 2.1.Were participants aware of their assigned intervention during the trial? | N |  |
|  | 2.2.Were carers and people delivering the interventions aware of participants' assigned intervention during the trial? | N |  |
|  | 2.3. If Y/PY/NI to 2.1 or 2.2: Were there deviations from the intended intervention that arose because of the experimental context? | NA |  |
|  | 2.4 If Y/PY to 2.3: Were these deviations likely to have affected the outcome? | NA |  |
|  | 2.5. If Y/PY/NI to 2.4: Were these deviations from intended intervention balanced between groups? | NA |  |
|  | 2.6 Was an appropriate analysis used to estimate the effect of assignment to intervention? | Y |  |
|  | 2.7 If N/PN/NI to 2.6: Was there potential for a substantial impact (on the result) of the failure to analyse participants in the group to which they were randomized? | NA |  |
|  | **Risk of bias judgement** | **Low** |  |
| **Bias due to missing outcome data** | 3.1 Were data for this outcome available for all, or nearly all, participants randomized? | Y |  |
|  | 3.2 If N/PN/NI to 3.1: Is there evidence that result was not biased by missing outcome data? | NA |  |
|  | 3.3 If N/PN to 3.2: Could missingness in the outcome depend on its true value? | NA |  |
|  | 3.4 If Y/PY/NI to 3.3: Is it likely that missingness in the outcome depended on its true value? | NA |  |
|  | **Risk of bias judgement** | **Low** |  |
| **Bias in measurement of the outcome** | 4.1 Was the method of measuring the outcome inappropriate? | N |  |
|  | 4.2 Could measurement or ascertainment of the outcome have differed between intervention groups? | PN |  |
|  | 4.3 Were outcome assessors aware of the intervention received by study participants? | N |  |
|  | 4.4 If Y/PY/NI to 4.3: Could assessment of the outcome have been influenced by knowledge of intervention received? | NA |  |
|  | 4.5 If Y/PY/NI to 4.4: Is it likely that assessment of the outcome was influenced by knowledge of intervention received? | NA |  |
|  | **Risk of bias judgement** | **Low** |  |
| **Bias in selection of the reported result** | 5.1 Were the data that produced this result analysed in accordance with a pre-specified analysis plan that was finalized before unblinded outcome data were available for analysis? | PN |  |
|  | 5.2 ... multiple eligible outcome measurements (e.g. scales, definitions, time points) within the outcome domain? | PN |  |
|  | 5.3 ... multiple eligible analyses of the data? | PY |  |
|  | **Risk of bias judgement** | **Some concerns** |  |
| **Overall bias** | **Risk of bias judgement** | **Some concerns** |  |

Supplementary 4: Appraisal of the included studies, using the GRADE tool

| Quality assessment | | | | | | | | Quality of evidence |  |
| --- | --- | --- | --- | --- | --- | --- | --- | --- | --- |
| No. of studies | Design | Risk of bias | Inconsistency | Indirectness | Imprecision | Publication bias | Other |  |  |
| Pain score on movement (early) | | | | | | | | |  |
| 5  (n = 386) | RCT | Serious^a^ | Serious^b^ | No serious indirectness | Serious^c^ | No serious publication bias | None | ⊕○○○  Very low |  |
| Pain score at rest (early) | | | | | | | | |  |
| 5  (n = 386) | RCT | Serious^a^ | No serious inconsistency | No serious indirectness | No serious imprecision | No serious publication bias | None | ⊕⊕⊕○  Moderate |  |
| Pain score on movement (12 h) | | | | | | | | |  |
| 4  (n = 296) | RCT | Serious^a^ | No serious inconsistency | No serious indirectness | No serious imprecision | No serious publication bias | None | ⊕⊕⊕○  Moderate |  |
| Pain score at rest (12 h) | | | | | | | | |  |
| 4  (n = 296) | RCT | Serious^a^ | No serious inconsistency | No serious indirectness | No serious imprecision | No serious publication bias | None | ⊕⊕⊕○  Moderate |  |
| Pain score on movement (24 h) | | | | | | | | |  |
| 5  (n = 376) | RCT | Serious^a^ | No serious inconsistency | No serious indirectness | No serious imprecision | No serious publication bias | None | ⊕⊕⊕○  Moderate |  |
| Pain score at rest (24 h) | | | | | | | | |  |
| 4  (n = 314) | RCT | Serious^a^ | Serious^b^ | No serious indirectness | Serious^c^ | No serious publication bias | None | ⊕○○○  Very low |  |
| Pain score on movement (late) | | | | | | | | | |
| 3  (n = 234) | RCT | Serious^a^ | No serious inconsistency | No serious indirectness | No serious imprecision | No serious publication bias | None | ⊕⊕⊕○  Moderate | |
| Pain score at rest (late) | | | | | | | | | |
| 3  (n = 234) | RCT | Serious^a^ | No serious inconsistency | No serious indirectness | No serious imprecision | No serious publication bias | None | ⊕⊕⊕○  Moderate | |
| Opioid consumption (24 h) | | | | | | | | | |
| 4  (n = 313) | RCT | Serious^a^ | Serious^b^ | No serious indirectness | Serious^c^ | No serious publication bias | None | ⊕○○○  Very low | |
| Post-operative nausea | | | | | | | | | |
| 5  (n = 373) | RCT | Serious^a^ | No serious inconsistency | No serious indirectness | Serious^d^ | No serious publication bias | None | ⊕⊕○○  Low | |
| Post-operative vomiting | | | | | | | | | |
| 4  (n = 300) | RCT | Serious^a^ | No serious inconsistency | No serious indirectness | Serious^d^ | No serious publication bias | None | ⊕⊕○○  Low | |

a. Serious risk of bias caused by inclusion of studies with some concerns (Abdelsalam 2024, Wang 2024, Şehirlioğlu 2025, Hay 2026).

b. Serious inconsistency caused by high heterogeneity (I² > 75%) and opposite effect directions across studies.

c. Serious imprecision caused by the re-expressed mean-difference confidence intervals crossing the minimal clinically important difference of 1 point (Myles et al. 2017).

d. Serious imprecision caused by risk ratio increase of 25% or more (Guyatt et al. 2011).

Supplementary 5: Results of the Systematic Review and Meta-analysis

1. Primary outcome: Pain at different time points

Figure S5-1.1a: Pain at rest (early), primary analysis

Figure S5-1.1b: Pain at rest (early), subgroup analysis (PENG+LFCN or PENG alone)

Figure S5-1.1c: Pain at rest (early), subgroup analysis (anterior versus lateral QLB)

Figure S5-1.1d: Pain at rest (early), sensitivity analysis

Figure S5-1.1e: Pain at rest (early), leave-one-out analysis

Figure S5-1.2a: Pain on movement (early), primary analysis

Figure S5-1.2b: Pain on movement (early), subgroup analysis (PENG+LFCN versus PENG alone)

Figure S5-1.2c: Pain on movement (early), subgroup analysis (anterior versus lateral QLB)

Figure S5-1.2d: Pain on movement (early), sensitivity analysis

Figure S5-1.2e: Pain on movement (early), leave-one-out analysis

Figure S5-1.3a: Pain at rest (12h), primary analysis

Figure S5-1.3b: Pain at rest (12h), subgroup analysis (PENG+LFCN versus PENG alone)

Figure S5-1.3c: Pain at rest (12h), subgroup analysis (anterior versus lateral QLB)

Figure S5-1.3d: Pain at rest (12h), sensitivity analysis

Figure S5-1.3e: Pain at rest (12h), leave-one-out analysis

Figure S5-1.4a: Pain on movement (12h), primary analysis

Figure S5-1.4b: Pain on movement (12h), subgroup analysis (PENG+LFCN versus PENG alone)

Figure S5-1.4c: Pain on movement (12h), subgroup analysis (anterior versus lateral QLB)

Figure S5-1.4d: Pain on movement (12h), sensitivity analysis

Figure S5-1.4e: Pain on movement (12h), leave-one-out analysis

Figure S5-1.5a: Pain at rest (24h), primary analysis

Figure S5-1.5b: Pain at rest (24h), subgroup analysis (PENG+LFCN versus PENG alone)

Figure S5-1.5c: Pain at rest (24h), subgroup analysis (anterior versus lateral QLB)

Figure S5-1.5d: Pain at rest (24h), sensitivity analysis

Figure S5-1.5e: Pain at rest (24h), leave-one-out analysis

Figure S5-1.6a: Pain on movement (24h), primary analysis

Figure S5-1.6b: Pain on movement (24h), subgroup analysis (PENG+LFCN versus PENG alone)

Figure S5-1.6c: Pain on movement (24h), subgroup analysis (anterior versus lateral QLB)

Figure S5-1.6d: Pain on movement (24h), sensitivity analysis

Figure S5-1.6e: Pain on movement (24h), leave-one-out analysis

Figure S5-1.7a: Pain at rest (late), primary analysis

Figure S5-1.7b: Pain at rest (late), subgroup analysis (PENG+LFCN versus PENG alone)

Figure S5-1.7c: Pain at rest (late), subgroup analysis (anterior versus lateral QLB)

Figure S5-1.7d: Pain at rest (late), sensitivity analysis

Figure S5-1.7e: Pain at rest (late), leave-one-out analysis

Figure S5-1.8a: Pain on movement (late), primary analysis

Figure S5-1.8b: Pain on movement (late), subgroup analysis (PENG+LFCN versus PENG alone)

Figure S5-1.8c: Pain on movement (late), subgroup analysis (anterior versus lateral QLB)

Figure S5-1.8d: Pain on movement (late), sensitivity analysis

Figure S5-1.8e: Pain on movement (late), leave-one-out analysis

2. Secondary Outcome: Opioid consumption, nausea and vomiting

Figure S5-2.1a: Opioid consumption (24h), primary analysis

Figure S5-2.1b: Opioid consumption (24h), subgroup analysis (PENG+LFCN versus PENG alone)

Figure S5-2.1c: Opioid consumption (24h), subgroup analysis (anterior versus lateral QLB)

Figure S5-2.1d: Opioid consumption (24h), sensitivity analysis

Figure S5-2.1e: Opioid consumption (24h), leave-one-out analysis

Figure S5-2.2a: Nausea, primary analysis

Figure S5-2.2b: Nausea, subgroup analysis (PENG+LFCN versus PENG alone)

Figure S5-2.2c: Nausea, sensitivity analysis

Figure S5-2.2d: Nausea, leave-one-out analysis

Figure S5-2.3a: Vomiting, primary analysis

Figure S5-2.3b: Vomiting, subgroup analysis (PENG+LFCN versus PENG alone)

Figure S5-2.3c: Vomiting, sensitivity analysis

Figure S5-2.3d: Vomiting, leave-one-out analysis

Supplementary 6: Adverse Events Reported by the Included Trials

| **Study** | **RoB** | **Adverse event** | **PENG (n/N)^a^** | **QLB (n/N)** | **p** |
| --- | --- | --- | --- | --- | --- |
| **Postoperative nausea^b^ (also meta-analyzed; Figure 4B)** | | | | | |
| Aslan 2025 | Low | Nausea | 8/40 | 10/40 | 0.59 |
| Et 2023 | Low | Nausea | 2/30 | 2/30 | 0.064 |
| Wang 2023 | Some concerns | Nausea | 12/45 | 14/45 | 0.642 |
| Abdelsalam 2024 | Some concerns | Nausea | 6/35 | 6/35 | 1.000 |
| Şehirlioğlu 2025 | Some concerns | Nausea | 0/43 | 2/30 | NR |
| Elshall 2025^c^ | Low | Nausea/vomiting | 0/31 | 0/31 | NR |
| **Postoperative vomiting^b^ (also meta-analyzed; Figure 4C)** | | | | | |
| Aslan 2025 | Low | Vomiting | 6/40 | 5/40 | 0.74 |
| Et 2023 | Low | Vomiting | 1/30 | 2/30 | 0.786 |
| Wang 2023 | Some concerns | Vomiting | 7/45 | 5/45 | 0.535 |
| Abdelsalam 2024 | Some concerns | Vomiting | 2/35 | 3/35 | 0.643 |
| **Transient quadriceps weakness** | | | | | |
| Aslan 2025 | Low | Quadriceps weakness, 6 h | 0/40 | 6/40 | 0.026 |
| Aslan 2025 | Low | Quadriceps weakness, 12 h and after | 0/40 | 0/40 | NR |
| Wang 2023 | Some concerns | Quadriceps weakness, any | 0/45 | 0/45 | NR |
| Şehirlioğlu 2025 | Some concerns | Quadriceps weakness, any | 0/43 | 0/30 | NR |
| **Other adverse events** | | | | | |
| Aslan 2025 | Low | Pruritus | 2/40 | 3/40 | 0.50 |
| Elshall 2025 | Low | Block failure | 0/31 | 0/31 | NR |
| **Falls** | | | | | |
| All trials | — | Falls | Not reported | Not reported | — |

a. Event counts are presented as the number of patients with the event over the number analyzed (n/N) in each arm.
b. Postoperative nausea and vomiting were pooled separately (nausea: RR 0.81, 95% CI 0.53–1.26, k=5; vomiting: RR 1.07, 95% CI 0.55–2.07, k=4); see Figure 4. Elshall et al. reported combined postoperative nausea and vomiting (0% in both block arms) and was therefore not included in the separate nausea and vomiting pools.

NR, not reported / not formally tested; RoB, risk of bias; —, not applicable.

Supplementary7: Re-expression of the pooled pain effects on a common 0–10 scale and interpretation against the minimal clinically important difference (MCID).

1. Supplementary Table S7-1

| **Pain outcome (window)** | **k** | **Primary SMD (95% CI)** | **Re-expressed MD  on 0–10 (95% CI)^b^** | **I²** | **Interpretation vs MCID 1 point^c^** |
| --- | --- | --- | --- | --- | --- |
| Pain at rest, early (~3 h) | 5 | -0.20 (-0.57 to 0.17) | -0.18 (-0.49 to 0.13) | 37 | Clinically important difference excluded |
| Pain on movement, early (~3 h) | 5 | -0.44 (-1.08 to 0.20) | **-0.49 (-1.36 to 0.38)** | 74 | Could not exclude a clinically important difference |
| Pain at rest, 12 h | 4 | 0.05 (-0.26 to 0.36) | 0.07 (-0.28 to 0.43) | 0 | Excluded |
| Pain on movement, 12 h | 4 | 0.03 (-0.33 to 0.39) | 0.01 (-0.48 to 0.50) | 0 | Excluded |
| Pain at rest, 24 h | 4 | -0.10 (-0.98 to 0.79) | **-0.14 (-1.41 to 1.14)** | 83 | Could not exclude a clinically important difference |
| Pain on movement, 24 h | 5 | 0.00 (-0.32 to 0.32) | -0.02 (-0.42 to 0.38) | 23 | Excluded |
| Pain at rest, late (48 h) | 3 | 0.13 (-0.01 to 0.28) | 0.16 (-0.20 to 0.52) | 0 | Excluded |
| Pain on movement, late (48 h) | 3 | 0.11 (-0.36 to 0.58) | 0.04 (-0.68 to 0.76) | 1 | Excluded |

All analyses used a random-effects model. A negative value favors the PENG block.

a. For interpretation of MCID, the same trials were re-expressed as mean differences on a common 0–10 pain scale (VAS 0–100 trials rescaled by dividing mean and SD by 10).
b. MCID = 1 point on the 0–10 scale. When the 95% CI of the mean difference lay entirely within ±1 point, a clinically important difference was excluded; when it extended beyond ±1 point, a clinically important difference could not be excluded.
2. Supplementary Figures: Re-expressed pooled analysis of pain at different time windows

Figure S7-2a: Re-analysis of the pain outcome on the scale 0 to 10 compared to MCID (1 point).

Figure S7-2b: Early resting pain sensitivity analysis re-expressed as a mean difference on the 0 to 10 scale

Figure S7-2c: Early motion pain, sensitivity analysis re-expressed as a mean difference on the 0 to 10 scale

Figure S7-2d: Twenty-four-hour rest-pain leave-one-out analysis re-expressed as a mean difference on the 0 to 10 scale
